# TLR7-MyD88-DC-CXCL16 axis results neutrophil activation to elicit inflammatory response in pustular psoriasis

**DOI:** 10.1038/s41419-023-05815-y

**Published:** 2023-05-09

**Authors:** Jiajing Lu, Xiaoyuan Zhong, Chunyuan Guo, Li Tang, Ning Yu, Chen Peng, Yangfeng Ding, Xunxia Bao, Jing Zhou, Yuling Shi

**Affiliations:** 1 Department of Dermatology, Shanghai Skin Disease Hospital, School of Medicine, Tongji University, Shanghai, 200443 China; 2 Institute of Psoriasis, School of Medicine, Tongji University, Shanghai, 200443 China; 3 School of Life Science, Anhui Medical University, Hefei, 230032 China

**Keywords:** Inflammation, Cell signalling

## Abstract

Pustular psoriasis (PP) is a chronic inflammatory disease associated with multiple complications, often with hyperthermia and hypoproteinemia, and its continued progression can be life-threatening. Toll-like receptor 7 (TLR7) induces dendritic cell (DC) production of inflammatory factors that exacerbate the inflammatory response in PP. A membrane-bound chemokine expressed on DCs, CXC motif chemokine ligand 16 (CXCL16) is overexpressed in PP lesions, and neutrophils express its receptor CXC chemokine receptor 6 (CXCR6). There are few studies on the PP immune microenvironment and it is unclear whether TLR7 and CXCL16 can be used as targets in PP therapy. Skin tissue (*n* = 5) and blood (*n* = 20) samples were collected from PP and healthy normal controls. The skin tissue transcriptome was analyzed to obtain the differentially expressed genes, and the immune microenvironment was deciphered using pathway enrichment. Tissue sequencing analysis indicated that TLR7, CXCL16, DCs, and neutrophils were involved in the PP process. The enzyme-linked immunosorbent assay, reverse transcription–PCR, and scoring table results demonstrated that TLR7 induced DC secretion of CXCL16, which enabled neutrophil activation of the secretion of the inflammatory factors interleukin-8 (IL-8) and tumor necrosis factor alpha (TNF-α). The co-culture of neutrophils with DCs treated with TLR7 inhibitor or TLR7 agonist demonstrated that TLR7 regulated neutrophil activation, migration, and apoptosis. We constructed imiquimod-induced psoriasis-like skin lesions in wild-type, *Cd11c-Cre Myd88*^*f/f*^, and *Mrp8-Cre Cxcr6*^*f/f*^ mice. The mouse models suggested that TLR7 might influence DC release of CXCL16 and neutrophil proinflammatory effects by interfering with the myeloid differentiation primary response gene 88 (MyD88) signaling pathway. In conclusion, the TLR7–MyD88–DC–CXCL16 axis is an important mechanism that promotes neutrophil migration to PP skin lesions and stimulates the inflammatory response.

## Background

Psoriasis is an autoimmune disease caused by the combined influence of polygenic genetic and environmental factors that seriously endangers patients’ physical and mental health, as it is a persistent condition that is difficult to control clinically. The worldwide prevalence of psoriasis is 2–3% [1]. Pustular psoriasis (PP) is a rare clinical psoriasis subtype that accounts for ~1.2% of the psoriasis population, with a complication rate of 17% and a mortality rate of 2% [2, 3]. There are few studies related to the PP immune microenvironment, which warrants further investigation.

It is generally accepted that an intricate crosstalk between infiltrating immune cells, activated keratin-forming cells, and dermal vascular cells mediate the major drivers of psoriasis. The role of T cells, dendritic cells (DCs), and macrophages has been extensively studied in psoriasis pathogenesis promotion by various immune cells and inflammatory factors [4, 5]. The most abundant cells in innate immunity, neutrophils are significantly elevated in neutrophils and in the neutrophil–lymphocyte ratio (NLR) in psoriasis patients [6]. Neutrophils secrete cytokines such as tumor necrosis factor alpha (TNF-α) and interleukin-8 (IL-8), and the chemotactic effect of these inflammatory factors leads to neutrophil infiltration in the psoriatic epidermis and promotes psoriasis inflammatory progression [7, 8]. There are fewer studies related to the progression of neutrophils and PP, but it is likely that neutrophils are an important cellular component in PP pathogenesis.

Toll-like receptor 7 (TLR7) is mainly expressed on DCs, monocytes, T lymphocytes, and B lymphocytes and has regulatory roles in immunodeficiency diseases and immune regulation [9, 10]. The synergistic action of TLR7 and imiquimod (IMQ, a TLR7 ligand) induces both the cytokines required to activate the Th17 pathway and the proinflammatory factor expression for psoriasis pathogenesis, and promotes IL-1, IL-2, and TNF-α production by DCs [11]. Currently, there are very few studies on TLR7 and PP progression, which warrants further examination.

CXC motif chemokine ligand 16 (CXCL16) is a membrane-bound chemokine expressed on DCs that binds specifically to CXC receptor 6 (CXCR6), promotes immune cell accumulation to inflammation sites, and plays a role in autoimmune diseases [12]. CXCL16 is overexpressed in the skin lesions of psoriasis patients [13]. CXCR6 can be expressed on neutrophils and the proinflammatory tumor microenvironment, leading to metastasis and poor prognosis of cancer [14, 15].

Based on these considerations, we investigated whether TLR7 can induce CXCL16 secretion by DCs by binding to CXCR6 on neutrophils, which would enable neutrophils to activate IL-8 and TNF-α secretion. The specific immune response induced by this mechanism might be responsible for initiating or exacerbating PP progression. The findings contribute to the deepening and refining of PP clinical immunotherapy and have profound implications for the development of more new drugs and therapies targeting PP and the study of other neutrophil-dominated inflammatory diseases.

## Results

### Tissue sequencing analysis indicated that TLR7, CXCL16, DCs, and neutrophils are involved in the PP process

**Fig. 1:**
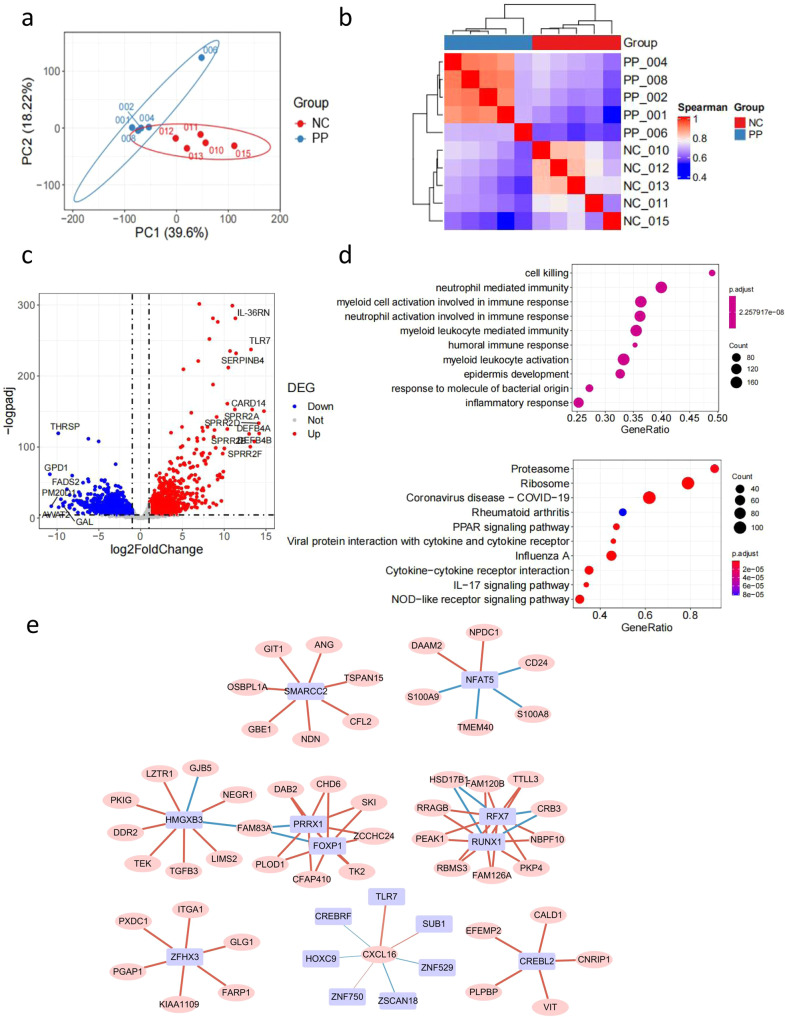
Transcriptome analysis revealed that TLR7, CXCL16, DCs, and neutrophils are involved in the PP process. **a**, **b** PCA and HCA results showing that the NC and PP samples were significantly different and that the samples clustered together within the groups. **c**, **d** DEG screening between groups and signaling pathway enrichment. **e** Co-network showing that CXCL16 was positively correlated with TLR7 expression.

The sequencing results of 10 skin tissue samples (five each from healthy normal controls [NC] and PP patients) were filtered according to the principle of Q30 > 75% and mapping rate > 5%. Principal component analysis (PCA) and hierarchical cluster analysis (HCA) clustering results demonstrated that the NC and PP samples were significantly different from each other and that the samples within the groups were clustered together (except for PP sample 006 which was abnormal, Fig. 1a). The nCount and nFeature statistics revealed that the variance of sample 006 was too large as compared with other samples within the PP group, so sample 006 was excluded. After sample and gene filtering, nine samples (PP: 001, 002, 004, 008; NC: 010, 011, 012, 013, 015) remained, and 11,460 genes were retained (Fig. 1b). The differentially expressed genes (DEGs, compared with the NC) were filtered according to the criteria of padj < 0.05 and logfc absolute value > 1, where 228 upregulated genes and 242 downregulated genes were obtained. The DEG volcano map demonstrated that *IL36RN*, *TLR7*, *SERPINB4*, *CARD14*, *DEFB4A*, *DEFB4B*, *SPRR2A*, *SPRR2B*, *SPRR2D*, and *SPRR2F* were highly expressed in the PP group. On the contrary, *THRSP*, *GPD1*, *FADS2*, *ANGPTL7*, *SEC14L6*, *GAL*, *ADIPOQ*, *PPP1R1A*, *AWAT2*, and *PM20D1* expression was low (Fig. 1c). The Gene Ontology biological process (GOBP) and Kyoto Encyclopedia of Genes and Genomes (KEGG) results suggested that the DEGs were mainly involved in immune pathways such as neutrophil-mediated immunity, myeloid cell activation, neutrophil activation, inflammatory response, and the PPAR and IL-17 signaling pathways (Fig. 1d). Transcription factors (TF) in the DEGs were screened, where the TF (purple) underwent Spearman correlation analysis with differentially expressed mRNA (pink), were filtered according to Benjamini-Hochberg (BH) < 0.05, and the top 100 TF–mRNA pairs with the largest correlation were plotted on a co-network. Blue lines indicated a negative correlation, red lines indicated a positive correlation, and thicker lines indicated a smaller *P*-value. The co-network indicated that CXCL16 positively correlated with TLR7 expression and was involved in the psoriasis process (Fig. 1e). The immune microenvironment was deciphered using RNA sequencing deconvolution, and the Wilcox test revealed that the PP group had significant activation of activated myeloid DCs, class-switched memory B cells, monocytes, neutrophils, plasmacytoid DCs, and CD4^+^ Th2. The proportion of CD4^+^ central memory T cells and stroma score were decreased significantly (Table 1).

**Table 1:** Transcriptome immune microenvironment analysis.

Cell	*p*	bh
Activated myeloid dendritic cell	0.0365	0.154111111
T cell CD4^+^ central memory	0.0342	0.154111111
Class-switched memory B cell	0.02	0.108571429
Monocyte	0.0195	0.108571429
Neutrophil	0.02	0.108571429
Plasmacytoid dendritic cell	0.02	0.108571429
T cell CD4^+^ Th2	0.0195	0.108571429
Stroma score	0.02	0.108571429

### TLR7 induces DC secretion of CXCL16, which enables neutrophil activation of IL-8 and TNF-α secretion

**Fig. 2:**
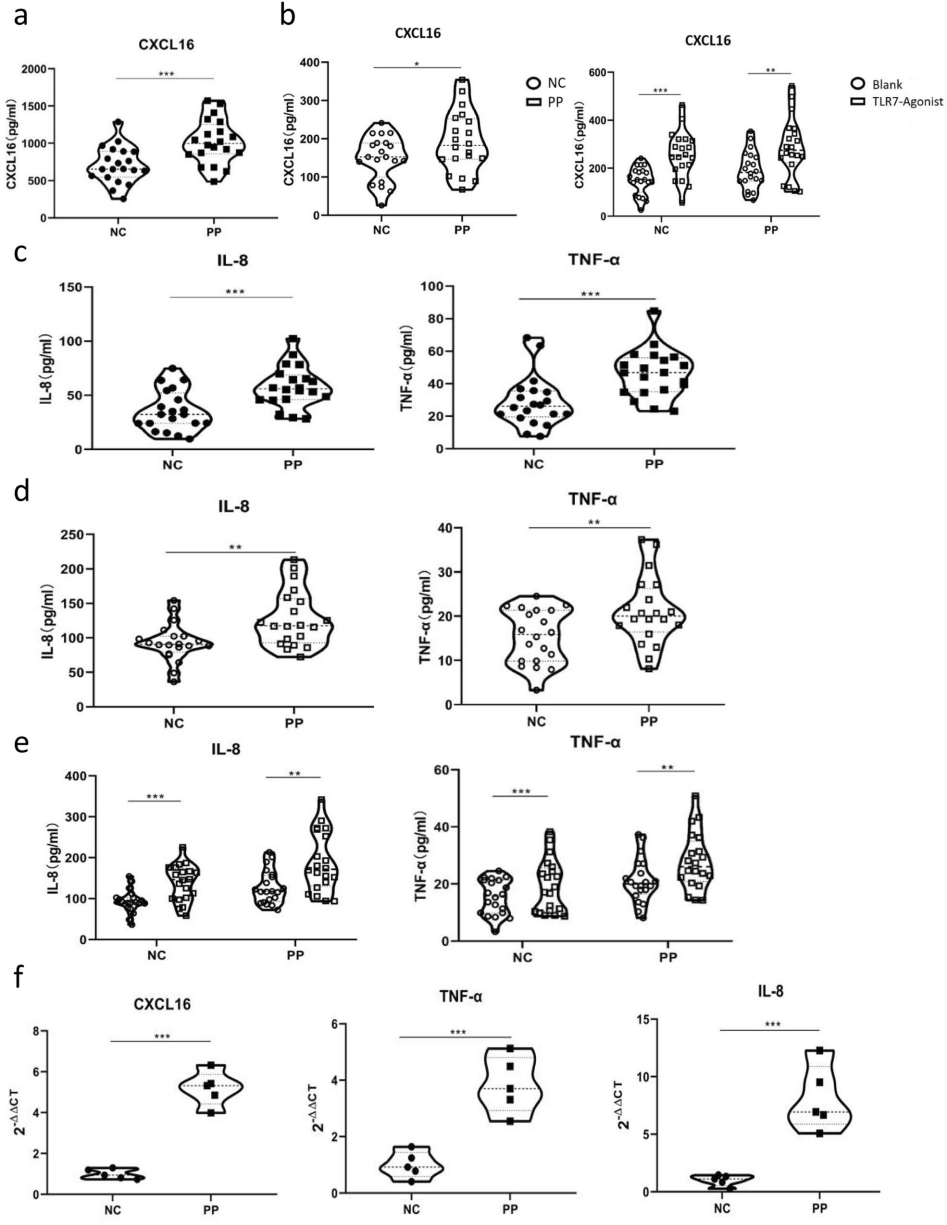
TLR7 induces DCs to secrete CXCL16, which allows neutrophils to activate IL-8 and TNF-α secretion. **a** ELISA detection showing that CXCL16 expression was significantly increased in the sera of PP patients (*n* = 20) as compared with the NC (*n* = 20). **b** CXCL16 expression was significantly increased in the PP group compared with the NC group before TLR7 agonist stimulation (*P < 0.05). DC stimulation by the TLR7 agonist significantly increased CXCL16 expression in both PP patients and NC. **c** IL-8 and TNF-α expression were most significantly increased in the PP group compared with the NC group. **d** IL-8 and TNF-α were increased before rhCXCL16 stimulation. **e** The most significant increase in IL-8 and TNF-α expression after rhCXCL16 stimulation. **f** RT-PCR results showing that CXCL16, IL-8, and TNF-α expression was most significantly higher in PP patients than in the NC. *P < 0.05, **P < 0.01, ***P < 0.001.

**Fig. 3:**
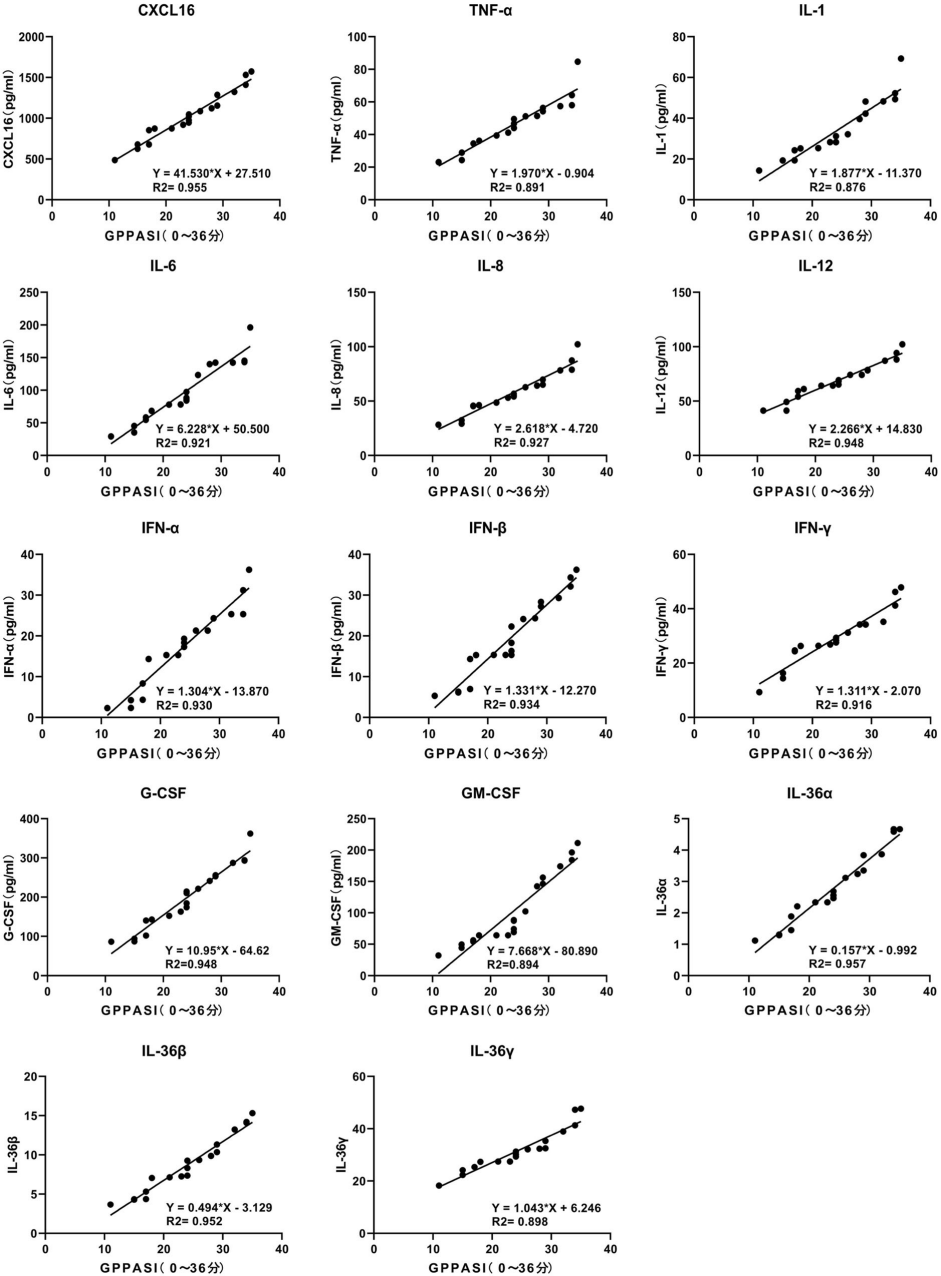
GPPASI score. Correlation analysis of GPPASI scores with CXCL16, TNF-α, IL-1, IL-6, IL-8, IL-12, IFN-α, IFN-β, IFN-γ, G-CSF, GM-CSF, IL-36α, IL-36β, and IL-36γ expression suggested a positive correlation (0 < r < 1).

Enzyme-linked immunosorbent assay (ELISA) measurement of CXCL16 expression in the sera of 20 PP patients and 20 NC suggested that CXCL16 expression was significantly increased in the PP patients as compared with the NC (*P* < 0.001, Fig. 2a). Peripheral blood DCs were isolated and cultured, stimulated by a TLR7 agonist, and the CXCL16 expression changes in the culture medium before and after stimulation were detected. The results suggested that the PP group had significantly increased CXCL16 expression compared with the NC group before TLR7 agonist stimulation (*P* < 0.05, Fig. 2b, left). DC stimulation by the TLR7 agonist significantly increased CXCL16 expression in both the PP patients and NC (*P* < 0.01, *P* < 0.001, Fig. 2b, right). ELISA detection determined that the PP group had significantly increased serum TNF-α, IL-1, IL-6, IL-8, IL-12, IFN-α, IFN-β, IFN-γ, G-CSF, GM-CSF, IL-36α, IL-36β, and IL-36γ expression levels compared with the NC group, where IL-8 and TNF-α expression was most significantly increased between the two groups (*P* < 0.05, *P* < 0.01, *P* < 0.001, Fig. 2c; Supplementary Fig. 1a depicts the changes in the levels of other inflammatory factors). The expression of all relevant inflammatory factors was increased before recombinant human (rh)CXCL16 stimulation (Fig. 2d). rhCXCL16 stimulation of neutrophils significantly increased the expression of all relevant inflammatory factors in both the PP patients and NC as compared to the pre-stimulation period. IL-8 and TNF-α expression after rhCXCL16 stimulation was most significantly increased between the two groups (*P* < 0.05, *P* < 0.01, *P* < 0.001, Fig. 2e; Supplementary Fig. 2a, b depict other inflammatory factor levels). Reverse transcription (RT)-PCR measurement determined that the expression of these inflammatory factors was significantly higher in the PP skin tissues as compared to the NC. Among them, CXCL16, IL-8, and TNF-α expression were most significantly increased (*P* < 0.05, *P* < 0.01, *P* < 0.001, Fig. 2f; Supplementary Fig. 1b depicts the changes in the levels of other inflammatory factors). Correlation analysis of the Generalized Pustular Psoriasis Area and Severity Index (GPPASI), Generalized Pustular Psoriasis Physician Global Assessment (GPPGA), Body Surface Area (BSA), and Dermatology Life Quality Index (DLQI) scores and neutrophil activation-based inflammatory factor expression in PP suggested a positive correlation (0 < r < 1, Fig. 3 demonstrates the GPPASI score; Supplementary Fig. 3 depicts the other three score types).

### TLR7 regulates intrinsic immune cell activation, migration, and apoptosis

**Fig. 4:**
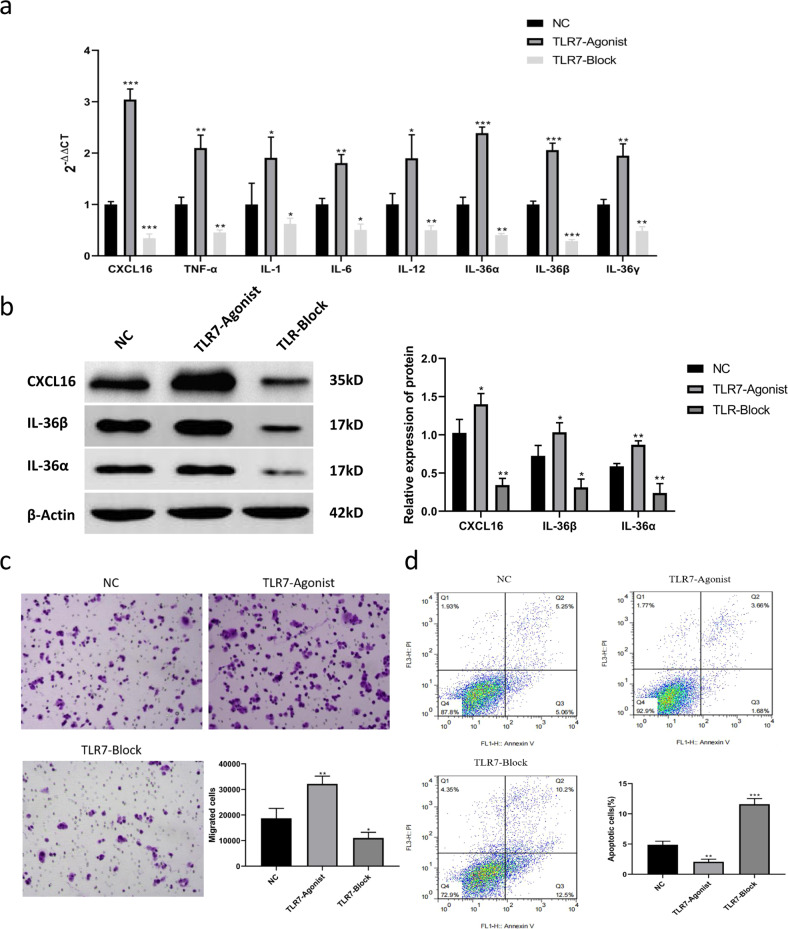
In vitro validation of the effect of TLR7-regulated CXCL16 expression on neutrophil activation, migration, and apoptosis. **a** RT-PCR detection of the expression changes of each intrinsic immune-related factor after TLR7 intervention in neutrophil and DC co-culture. CXCL16, IL-36α, and IL-36β expression was most significantly changed. **b** Western blot detection and analysis of CXCL16, IL-36α, and IL-36β expression changes before and after TLR7 intervention. **c** Transwell assay demonstrating that the TLR7 inhibitor inhibited neutrophil migration and that the TLR7 agonist promoted it. **d** TLR7 intervention after neutrophil and DC co-culture, where the neutrophil apoptosis rate was detected and analyzed by flow cytometry. *P < 0.05, **P < 0.01, ***P < 0.001.

DCs treated with TLR7 inhibitor or TLR7 agonist were co-cultured with neutrophils. The RT-PCR results revealed that the TLR7 agonist significantly increased the expression of genes such as the DC-related factors (CXCL16, TNF-α, IL-1, IL-6, IL-12) and neutrophil activation-related factors (IL-36α, IL-36β, IL-36γ), while the TLR7 inhibitor yielded opposite results (Fig. 4a). The effect of TLR7 on the protein expression levels of these factors was verified by western blotting, which yielded results that were consistent with that of RT-PCR (Fig. 4b).

The Transwell assay results suggested that the TLR7 agonist promoted neutrophil migration, while inhibiting TLR7 mediated the opposite effect (*P* < 0.05, *P* < 0.01, Fig. 4c). Flow cytometry observation revealed that the neutrophil apoptosis rate was 4.99 ± 1.06% in the NC group, 12.51 ± 2.13% in the TLR7 inhibitor group, and 2.21 ± 0.57% in the TLR7 agonist group (*P* < 0.01, *P* < 0.001. Fig. 4d).

### Animal models suggest that TLR7 may influence DC release of CXCL16 and neutrophil proinflammatory effects by interfering with the MyD88 signaling pathway

**Fig. 5:**
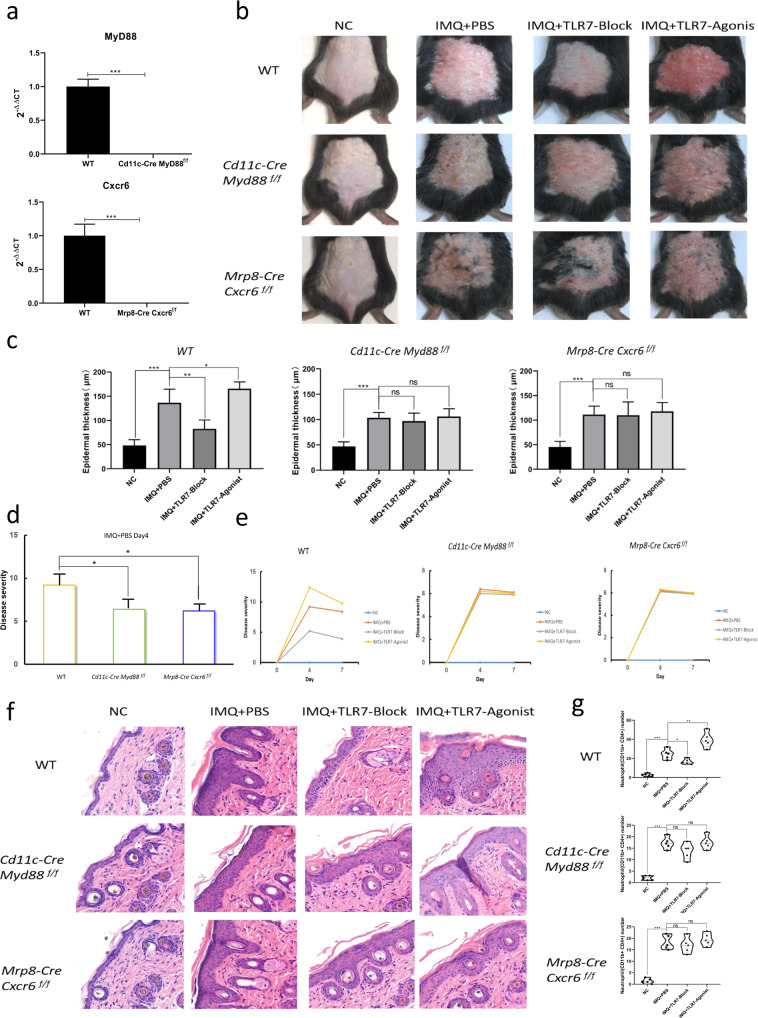
Morphological and neutrophil number changes of skin lesions in three psoriasis-like mouse models. **a** qPCR detection of MyD88 and CXCR6 knockout in mouse DCs and neutrophils. **b** Appearance of the three mouse models of psoriasis. **c** Detection of epidermal thickness before and after TLR7 intervention in the mouse models. **d** Comparison of PASI scores of the mouse models on modeling day 4. **e** Comparison of PASI scores before and after TLR7 intervention in the mouse models. **f** HE staining before and after establishment of the mouse models with TLR7 intervention. **g** Number of activated neutrophils in the mouse models after TLR7 inhibitor or TLR7 agonist treatment. *P < 0.05, **P < 0.01, ***P < 0.001.

DCs and neutrophils from wild-type (WT), Cd11c-Cre, and MyD88 f/f mice were obtained to detect the effect of knocking out MyD88 and CXCR6. The quantitative PCR (qPCR) results indicated successful construction of *Cd11c-Cre Myd88*^*f/f*^ and *Mrp8-Cre Cxcr6*^*f/f*^ knockout mice (Fig. 5a).

We constructed IMQ-induced psoriasis-like skin lesions in the WT, *Cd11c-Cre Myd88*^*f/f*^, and *Mrp8-Cre Cxcr6*^*f/f*^ mice. After 4 days of modeling, the WT IMQ + PBS group exhibited significantly worse lesions compared with the NC group, while lesions were reduced in the IMQ + TLR7 inhibitor group compared with the IMQ + PBS group and were increased in the IMQ + TLR7 agonist group, with more significant diffuse erythema. The *Cd11c-Cre Myd88*^*f/f*^ and *Mrp8-Cre Cxcr6*^*f/f*^ IMQ + PBS groups had aggravated skin lesions compared with the NC group, but there were no significant group differences in the severity of skin lesions in the IMQ + TLR7 inhibitor, IMQ + TLR7 agonist, and IMQ + PBS groups (Fig. 5b).

Examination and statistical analysis of the epidermal thickness of the three mouse models of psoriasis revealed significantly increased epidermal thickness in the IMQ + PBS group compared with the NC group, where the histopathological manifestations of lesions in the IMQ + PBS group were hyperkeratosis, hyperkeratosis, spinous layer thickening, and fungal inflammatory cell infiltration, which were consistent with the histological manifestations of psoriatic lesions. The WT IMQ + TLR7 inhibitor group demonstrated spinous layer thinning as compared to the IMQ + PBS group, with partial restoration of normal epidermal protrusions and reduced dermal inflammatory cell infiltration, while the IMQ + TLR7 agonist group demonstrated significant pinous layer thickening. However, there were no statistically significant differences between the *Cd11c-Cre Myd88*^*f/f*^ and *Mrp8-Cre Cxcr6*^*f/f*^ knockout mice. The WT mice demonstrated more pronounced skin lesion thickening than the other two mouse models (Fig. 5c, f).

The Psoriasis Area and Severity Index (PASI) scores for skin lesion severity in the three mouse models suggested that the PASI scores on day 4 of IMQ modeling in the WT mice were significantly higher than those in the other two groups (*P* < 0.05, Fig. 4d). The PASI scores of the WT mice demonstrated a decreasing trend after TLR7 inhibitor intervention and a significant increasing trend after TLR7 agonist stimulation. In contrast, the other two groups demonstrated no differential changes in PASI scores before and after TLR7 intervention (Fig. 5e).

**Fig. 6:**
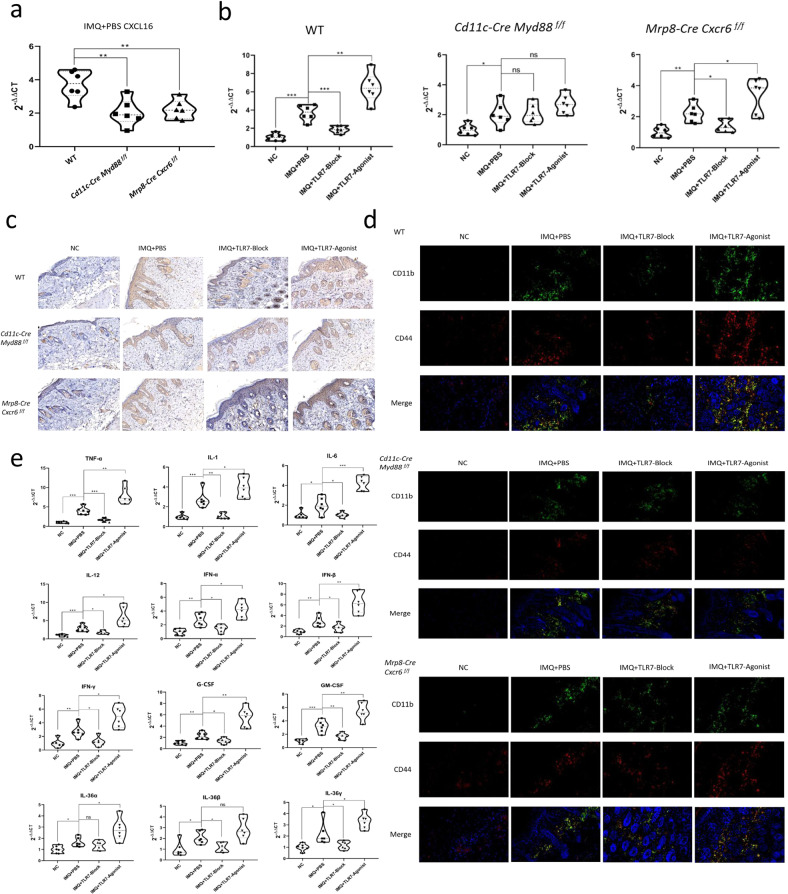
TLR7 might affect CXCL16 release from DCs and neutrophil proinflammatory effects by interfering with the MyD88 signaling pathway. **a** RT-PCR detection of *CXCL16* gene expression in the IMQ + PBS groups. **b** RT-PCR detection of *CXCL16* gene expression changes in the mouse models before and after TLR7 intervention. **c** IHC detection of CXCL16 expression in the mouse models before and after TLR7 intervention. **d** Immunofluorescence detection of CD11b (green) and CD44 (red) expression in the mouse models. **e** RT-PCR detection of the expression changes of each factor in the IMQ-induced WT mouse model before and after TLR7 intervention. *P < 0.05, **P < 0.01, ***P < 0.001.

In all three psoriasis mouse models, neutrophil number and immunofluorescence (CD11b and CD44) and CXCL16 expression were increased to different degrees in the IMQ + PBS group as compared to the NC group. The intensity of CXCL16, CD11b, and CD44 expression and activated neutrophil numbers were more significantly increased in the WT IMQ + PBS group compared to the other two mouse models (*P* < 0.01, Figs. 5g, 6a, d). The WT IMQ + TLR7 inhibitor group had significantly reduced CXCL16 expression and neutrophils compared to the IMQ + PBS group, whereas these parameters were significantly increased in the IMQ + TLR7 agonist group as compared to the IMQ + PBS group. The *Mrp8-Cre Cxcr6*^*f/f*^ knockout mice demonstrated the same CXCL16 expression trend as the WT mice, but TLR7 did not affect the neutrophil number and CD11b and CD44 expression. Furthermore, TLR7 did not affect CXCL16, CD11b, and CD44 expression and neutrophil numbers in the *Cd11c-Cre Myd88*^*f/f*^ knockout mice (Fig. 6b–d).

**Fig. 7:**
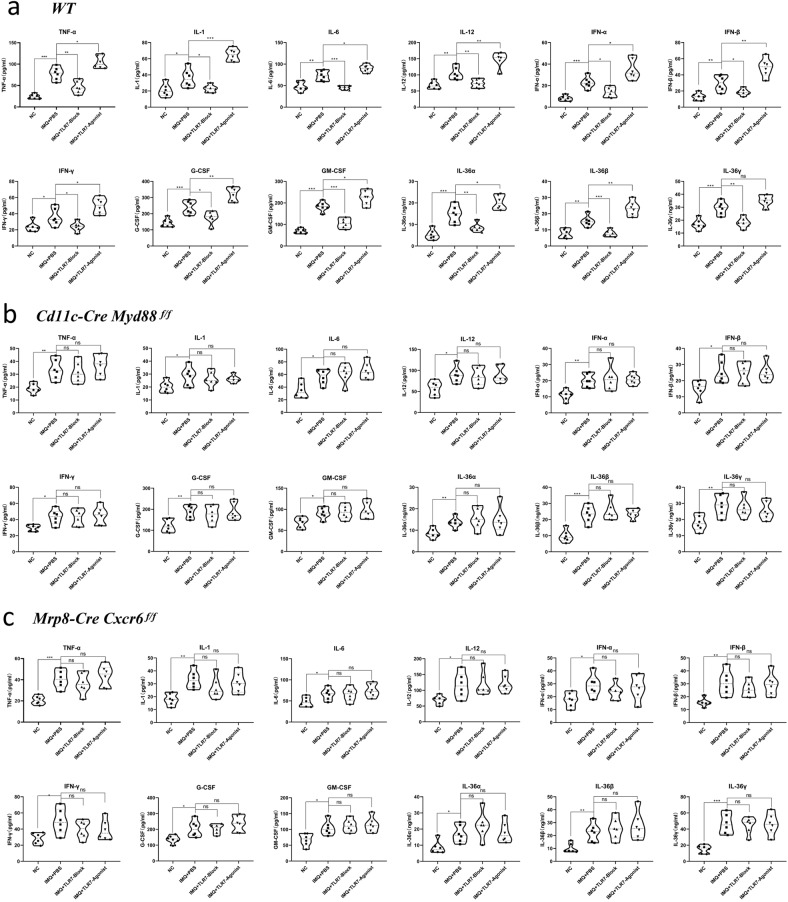
Inflammatory cytokine levels in mouse serum after TLR7 blockage or agonist treatment. **a** WT group. **b** *Cd11c-Cre Myd88*^*f/f*^ group. **c** *Mrp8-Cre Cxcr6*^*f/f*^ group.

Skin tissue RT-PCR detection determined that the IMQ + PBS groups in all three psoriasis mouse models had significantly increased TNF-α, IL-1, IL-6, IL-12, IFN-α, IFN-β, IFN-γ, G-CSF, GM-CSF, IL-36α, IL-36β, and IL-36γ gene expression compared to the NC groups. In the WT mice, the expression of each factor was significantly reduced in the IMQ + TLR7 inhibitor group compared to the IMQ + PBS group (except IL-36α) and was significantly increased in the IMQ + TLR7 agonist group compared to the IMQ + PBS group (except IL-36β) (*P* < 0.05, *P* < 0.01, *P* < 0.001, Fig. 6e). The expression of each factor in the other two models was not significantly different between the IMQ + PBS, IMQ + TLR7 inhibitor, and IMQ + TLR7 agonist groups (*P* > 0.05). There was a decreasing trend in the expression of all factors compared to the IMQ-induced WT psoriasis-like mouse model (Supplementary Fig. 4a, b). Measurement of the inflammatory cytokine levels in the mouse serum (Fig. 7) revealed that the expression of each factor was significantly reduced in the WT IMQ + TLR7 inhibitor group compared to the IMQ + PBS group and was significantly increased in the IMQ + TLR7 agonist group. In the *Mrp8-Cre Cxcr6*^*f/f*^ and *Cd11c-Cre Myd88*^*f/f*^ knockout mice, the expression of each inflammatory factor was significantly increased in the IMQ + PBS group as compared to the NC group, but was not affected by TLR7 inhibitor or TLR7 agonist.

## Discussion

As a chronic inflammatory disease, PP is associated with multiple comorbidities, often with hyperthermia and hypoproteinemia, and its continued progression can be life-threatening. PP pathogenesis is complex and it is generally believed that the main drivers are excessive inflammatory and chemokine activation in the PP skin lesions, which leads to immune cell infiltration and excessive proliferation of keratin-forming cells.

DCs are important antigen-presenting cells of the immune system that effectively recognize pathogens through pattern recognition receptors, which include TLRs, and the secretion of diverse factors to activate other immune cells [16]. TLR7 is expressed on DCs and is important in psoriasis development as an intracellular TLR that mediates viral nucleic acid recognition [17]. Rutaecarpine inhibited IMQ-induced psoriasis-like dermatitis by inhibiting the NF-κB and TLR7 pathways in mice [18]. TLR7 antagonists demonstrated promising results in psoriasis treatment [19]. It has also been suggested that psoriatic lesions contain numerous TLR7-expressing plasmacytoid DC infiltrates and that upon binding to its ligand, TLR7 induces DC-regulated immune responses, which promote the release of inflammatory mediators and cytokines (e.g., IL-8, TNF-α) and contribute to the inflammatory response in psoriasis mainly through the MyD88-dependent signaling pathway. This study is one of the few to demonstrate the therapeutic effect and possible pathway of TLR7 in PP.

PP is an aseptic inflammatory disease. However, the exposure of chronically poorly healed lesions to complex microbial environments causes rapid and massive overload production of cytokines, such as TNF-α, IL-1, IL-6, IL-8, IL-12, IFN-α, IFN-β, IFN-γ, G-CSF, GM-CSF, and IL-36 [20–23]. In this study, we demonstrated the presence of neutrophil activation-related factors (IL-8, G-CSF, GM-CSF, IL-36α, IL-36β, and IL-36γ), other innate immunity-related factors (CXCL16, TNF-α, IL-1, IL-6, and IL-12), and adaptive immunity-related factors (IFN-α, IFN-β, and IFN) in PP lesions. The high expression of these factors was positively correlated with disease severity, and intrinsic immunity played a dominant role. It has been hypothesized that these cytokines will in turn act on DCs to induce a specific immune response, leading to further epidermal cell proliferation and abnormal differentiation. This process will also express neutrophil chemokines that mobilize and recruit more neutrophils to the lesion, causing massive inflammatory cell infiltration in the lesion, where inflammatory cell migration is precisely regulated by a complex network of chemokines, such as CXCL16. CXCR6 is a specific and unique CXCL16 receptor on neutrophils, and CXCL16 binding to CXCR6 drives immune cells to the inflammation site, thereby possibly promoting PP development [24–28]. CXCL16 was dependent on TLR2/4 and MyD88 signaling for neutrophil recruitment in the cerebrospinal fluid of patients and mice with pneumococcal meningitis [14]. However, no correlation between TLR7 and CXCL16 has been reported to date. Based on the above results, we speculate that TLR7 regulates CXCL16 expression via the MyD88 pathway, which chemotactically activates neutrophils and causes high expression of the associated inflammatory factors, inducing an innate immune response. TLR7 regulates the activation, migration, and apoptosis of intrinsic immune cells such as neutrophils, and the TLR7–MyD88–DC–CXCL16–CXCR6–neutrophil axis is important in disease development in animal models. Blocking this pathogenic axis is expected to control PP progression.

Neutrophils constitute the most abundant leukocyte type in peripheral blood, where they invade the stratum corneum and secrete neutrophil extracellular traps, causing inflammation. Neutrophils are important immune effector cells in the psoriasis process [29]. Neutrophils link the innate immune system (macrophages, DCs) to the adaptive immune system (T cells), forming a complex network of immune responses and secreting the enzymes and proinflammatory mediators involved in the psoriasis pathophysiological process [30]. In the present study, we demonstrated that neutrophils are activated in PP patients, leading to elevated levels of IL-8 and TNF-α, which exacerbate skin damage. The use of infliximab against TNF-α proved to be an effective therapy in clinical trials for treating severe PP, as evidenced by the downregulation of pro-disease chemokines such as IL-8, GRO-α, and MCP-1 [31]. CXCL16 mediates neutrophil migration to the epidermis at the lesion and enhances TNF-α and IL-8 release. Therefore, it is an important and promising target for PP therapy.

The limitation of this paper is that the IMQ-induced mouse model does not fully reflect PP. As there is no clear method to trigger pustular lesion development in animal models, we used conventional IMQ-induced psoriasis mice. Given this limitation, much time and effort should be invested to screen suitable methods, and point mutant mice might be a promising PP modeling organism [32]; nevertheless, further research is still needed. In addition, the number of skin samples undergoing transcriptome sequencing was not very large due to the low incidence of PP. We will attempt to obtain informed consent from more patients in the future to expand the sample size.

## Conclusion

The TLR7–MyD88–DC–CXCL16 axis is an important mechanism that promotes neutrophil migration to PP skin lesions and stimulates the inflammatory response. This research provided a more comprehensive and in-depth understanding of the intrinsic immune response in PP, which is expected to fundamentally block neutrophil chemotaxis activation and thereby inhibit the secretion of large amounts of inflammatory factors, providing a scientific and theoretical basis for targeted and precise treatment of the disease.

## Methods

### Patients and samples

Skin tissue (*n* = 5) and blood samples (*n* = 20) were collected from clinically and histologically verified PP patients at our hospital who did not have other systemic diseases or autoimmune disorders and who were not treated with immunosuppressive drugs within 1 month. NC skin (*n* = 5) and blood (*n* = 20) were obtained from donors who had undergone excision of pigmented nevi at our dermatological surgery department. The Shanghai Skin Disease Hospital Ethics Committee approved the study protocol and informed consent was obtained from the participants.

Inclusion criteria: (1) age 18–65 years, regardless of gender; (2) confirmed diagnosis of PP; (3) disease duration ≥ 6 months; (4) voluntarily signed the informed consent form.

Exclusion criteria: (1) presence of other types of psoriasis; (2) presence of severe, uncontrolled, progressive organ and systemic diseases, malignancies, and other autoimmune diseases; (3) presence of type I diabetes mellitus or refractory type II diabetes mellitus; (4) pregnant or breastfeeding.

### Sequencing analysis of skin tissue samples

RNA was extracted from the PP and NC skin tissue samples, reverse-transcribed into complementary DNA, then PCR-amplified. The amplified products were purified and quality-controlled, then subjected to HiSeq PE150 sequencing. The samples were subjected to PCA and HCA, and filtered and analyzed for DEGs using *P* < 0.05 as a criterion. GOBP and KEGG analysis were conducted. The DEG TF were filtered and the TF underwent Spearman correlation analysis with differentially expressed mRNAs filtered according to BH < 0.05. Co-expression networks were constructed for the top 100 TF–mRNA pairs with the greatest correlation. The immune microenvironment was calculated for transcriptome data, and the Wilcox test was performed.

### RNA isolation and RT-qPCR

TRIzol (1 ml) was added in a 1.5-ml centrifuge tube, mixed well, and placed on ice for 5 min. Chloroform (200 µl) was added to a clean 1.5-ml centrifuge tube, to which an equal volume of isopropanol from an ice bath was added. Then, the tube was inverted and shaken to mix well, incubated at −20 °C for >10 min, then centrifuged at 4 °C and 12,000 × *g* for 10 min. The supernatant was discarded, 1 ml 75% ethanol was added in an ice bath, the centrifuge tube was inverted to mix, then the sample was vortexed and centrifuged at 4 °C for 5 min at 12,000 × *g*. The supernatant was removed and the precipitate was dried in a cool place (no more than 3 min). The RNA was lysed with 30–50 µl diethyl pyrocarbonate (DEPC) water. qPCR was performed after reverse transcription according to the kit instructions.

### Isolation of DCs from peripheral blood

The blood samples were diluted 1:1 with phosphate-buffered saline (PBS) culture medium. A 15-ml centrifuge tube was pre-filled with lymphocyte isolate and the diluted blood was carefully added on top so that the isolate:PBS:fresh blood ratio was 3:1:1. The preparation was centrifuged at room temperature at 720 × *g* for 25 min. Then, the centrifuge tube was removed, the white layer carefully aspirated, resuspended in 5× PBS volume, mixed well, and centrifuged at 500 × *g* for 10 min at room temperature. The supernatant was discarded, 1 ml PBS was added, mixed well, transferred to a 1.5 ml Eppendorf tube, and centrifuged at 300 × *g* for 5 min at room temperature. The supernatant was discarded and the cells were resuspended in RPMI 1640 complete medium to which 1000 U/ml GM-CSF and 1000 U/ml IL-4 were added. The medium was changed every 2–3 days and 200 U/ml TNF-α was added on day 6. The cells were incubated for 2–6 days, where some cells were removed for phenotypic analysis. CD1c and CD86 flow antibody was added to the removed cells for staining and labeling, followed by flow cytometry analysis.

### Isolation of neutrophils from peripheral blood

After centrifugation, the centrifuge tube contained two layers of milky white cells, where the upper layer was a single nuclear cell layer and the lower layer comprised neutrophils. The neutrophil layer was carefully aspirated from the separation solution with a pipette and transferred to another 15-ml centrifuge tube. Washing solution (10 ml) was added to the tube, mixed and washed, and centrifuged at room temperature for 10 min at 500 × *g*. The supernatant was discarded, and the resulting cells were resuspended in 5 ml washing solution using a pipette and centrifuged for 10 min at 500 × *g*. The cells were resuspended in PBS and counted for Diff-Quik staining. CD44 and CD11b flow antibody was added to the removed cells for staining and labeling, followed by flow cytometry analysis.

### ELISA

The relative expression of TNF-α, IL-1, IL-6, IL-8, IL-12, IFN-α, IFN-β, IFN-γ, G-CSF, GM-CSF, IL-36α, IL-36β, and IL-36γ in the serum samples was detected using an enzyme marker according to the reagent manufacturer’s instructions.

### Scoring table

Supplementary Tables 1, 2, 3, and 4 list the GPPASI, GPPGA, BSA, and DLQI scoring rules, respectively.

### Psoriasis animal model

Twelve 6-week-old C57BL/6 mice were randomly divided into four groups (NC, PBS, TLR7 agonist, TLR7 inhibitor) containing three mice each, and the dorsal region was dehaired. The NC group did not undergo any treatment after back hair removal. IMQ ointment (5%) was applied to the backs of the mice in the other three groups. After 2 h, each group was treated with PBS, TLR7 agonist (T4258, Topscience), or TLR7 inhibitor (HY-124603, MCE). The mice were housed in separate cages in a specific pathogen-free (SPF) environment and killed after 10 days. Dorsal lesions were obtained for hematoxylin–eosin (HE) staining (to identify abnormal pathological changes in the tissue) and immunohistochemistry (IHC, to detect CXCL16 secretion). The Shanghai Skin Disease Hospital Ethics Committee approved the study protocol.

### Establishment and intervention of IMQ-induced psoriasis-like mouse model

Twenty-four WT mice (6-weeks old, SPF) were randomly selected and divided into NC, PBS, TLR7 inhibitor, TLR7 agonist groups, each containing six mice. *Cd11c-Cre Myd88*^*f/f*^ and *Mrp8-Cre Cxcr6*^*f/f*^ knockout mice were also grouped in the same manner.

Hair was removed from a 2 cm × 3 cm area on the backs of the mice. In the experimental group, 62.5 mg IMQ cream was applied to the dehaired mouse backs. Subsequently, skin thickening and scaly erythema appeared on the area for 7 consecutive days. Pathological examination of the skin revealed results consistent with psoriasis-like changes. In the NC group, equal amounts of petroleum jelly were applied to the same area at the same time and frequency.

In the TLR7 agonist and inhibitor groups, the mice were gavaged with 3 mg/kg/day TLR7 agonist (loxoribine) or 20 mg/kg/day TLR7 inhibitor (hydroxychloroquine) 4 h prior to IMQ cream application and were maintained for 7 days.

### HE staining

Mouse skin lesions or control skin tissues were sampled for subsequent experiments. One part of the obtained tissues was preserved in formalin for subsequent pathological staining while the second part was immediately stored at −80 °C for subsequent experiments such as PCR. The tissues were paraffin-embedded, sectioned, HE-stained, and sealed using neutral gum. The stained sections were examined microscopically under a light microscope, and images were captured to analyze the staining results.

### Immunofluorescence staining

Tissue sections were fixed in 4% paraformaldehyde for ~20 min, then washed three times with PBS. Next, the sections were permeabilized with 0.2% Triton X-100 for ~10 min and washed three times with PBS. Subsequently, the sections were blocked using the same host serum as the secondary antibody for ~30 min, followed by three washes with PBS. Then, the primary antibody was added and incubated at 4 °C overnight, followed by three washes with PBS. The next day, the sections were incubated with the secondary antibody for 2 h at room temperature protected from light, followed by three washes in PBS. The nuclei were stained with DAPI, then direct fluorescence images were captured. Finally, the PBS was washed off with distilled water and the tissue was sealed with glycerol. The detection index was CD11b and CD44 immunofluorescence double staining. Finally, the stained sections were photographed and analyzed.

### Neutrophil and DC co-culture

The isolated DCs and neutrophils were inoculated in a Transwell system (6-well, 0.4-μm pore size), and 90 nM TLR7 agonist or 10 µM TLR7 inhibitor was added to the experimental groups and incubated in a cell incubator. After 24 h, the co-cultured neutrophils were collected for PCR, western blotting, and flow cytometry assays.

### Statistical analysis

Statistics analyses were performed using Prism 8 (GraphPad, San Diego, USA) and significance is indicated in the results (*P < 0.05, **P < 0.01, ***P < 0.001). Data were expressed as means±standard deviation (SD). Western blots were quantified by densitometric analyses using ImageJ software. Differences between two groups were carried out using Student’s *t* test. *P*-value < 0.05 was considered statistically significant. Mice were randomly allocated to experimental groups.

## Supplementary information

Supplementary figure 1

Supplementary figure 2

Supplementary figure 3

Supplementary figure 4

Supplementary table 1

Supplementary table 2

Supplementary table 3

Supplementary table 4

orginal western blots-ACTIN

orginal western blots-CXCL16

orginal western blots-IL-36α

orginal western blots-IL-36β

aj-checklist

## Data Availability

The datasets used and/or analyzed during the current study are available from the corresponding author on reasonable request.
