# Supplementary figures and images for: TLR7-MyD88-DC-CXCL16 axis results neutrophil activation to elicit inflammatory response in pustular psoriasis
Source: Cell Death Dis. 2023 May 9;14(5):315. doi: 10.1038/s41419-023-05815-y (PMC10170143; doi:10.1038/s41419-023-05815-y)

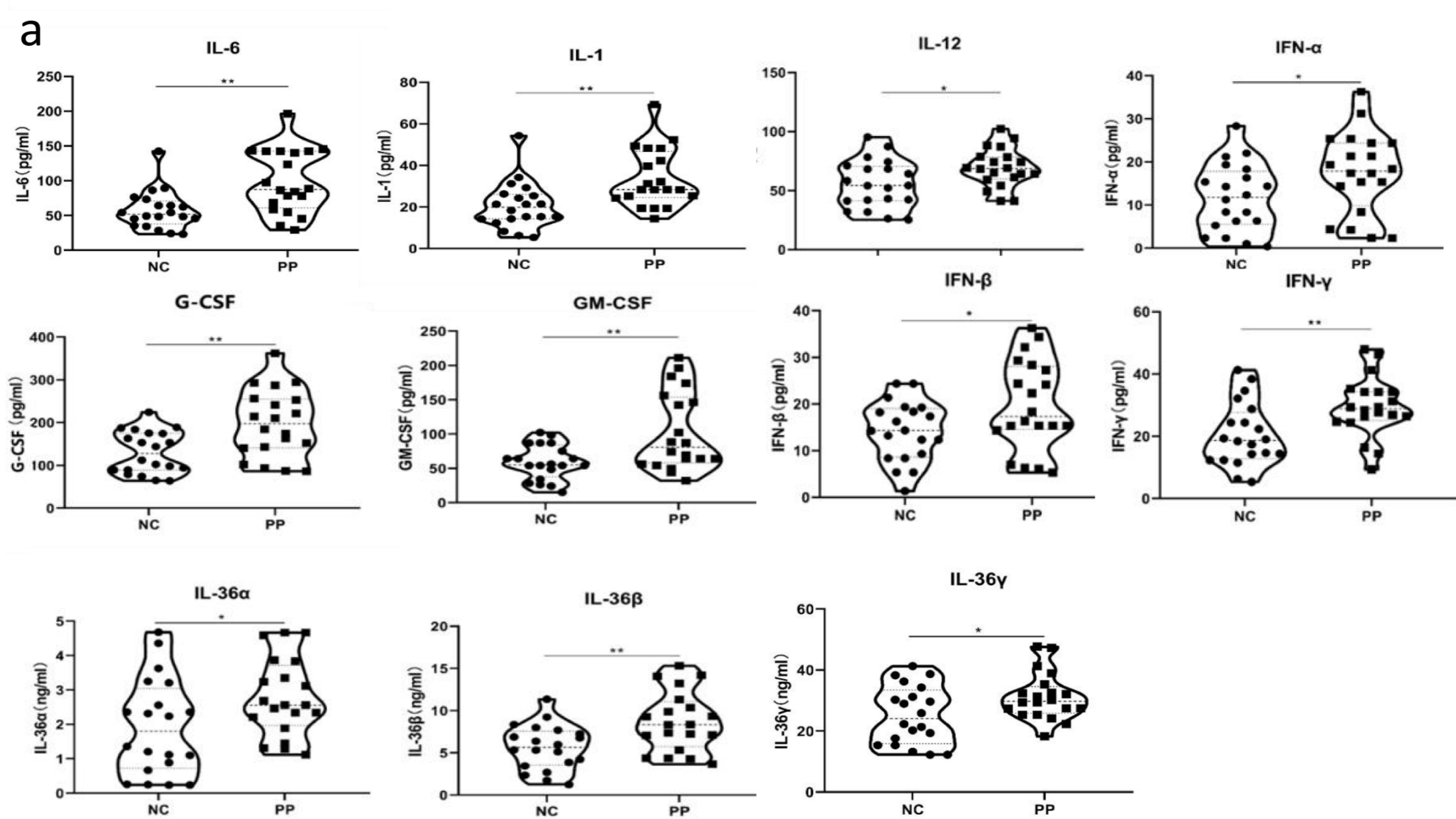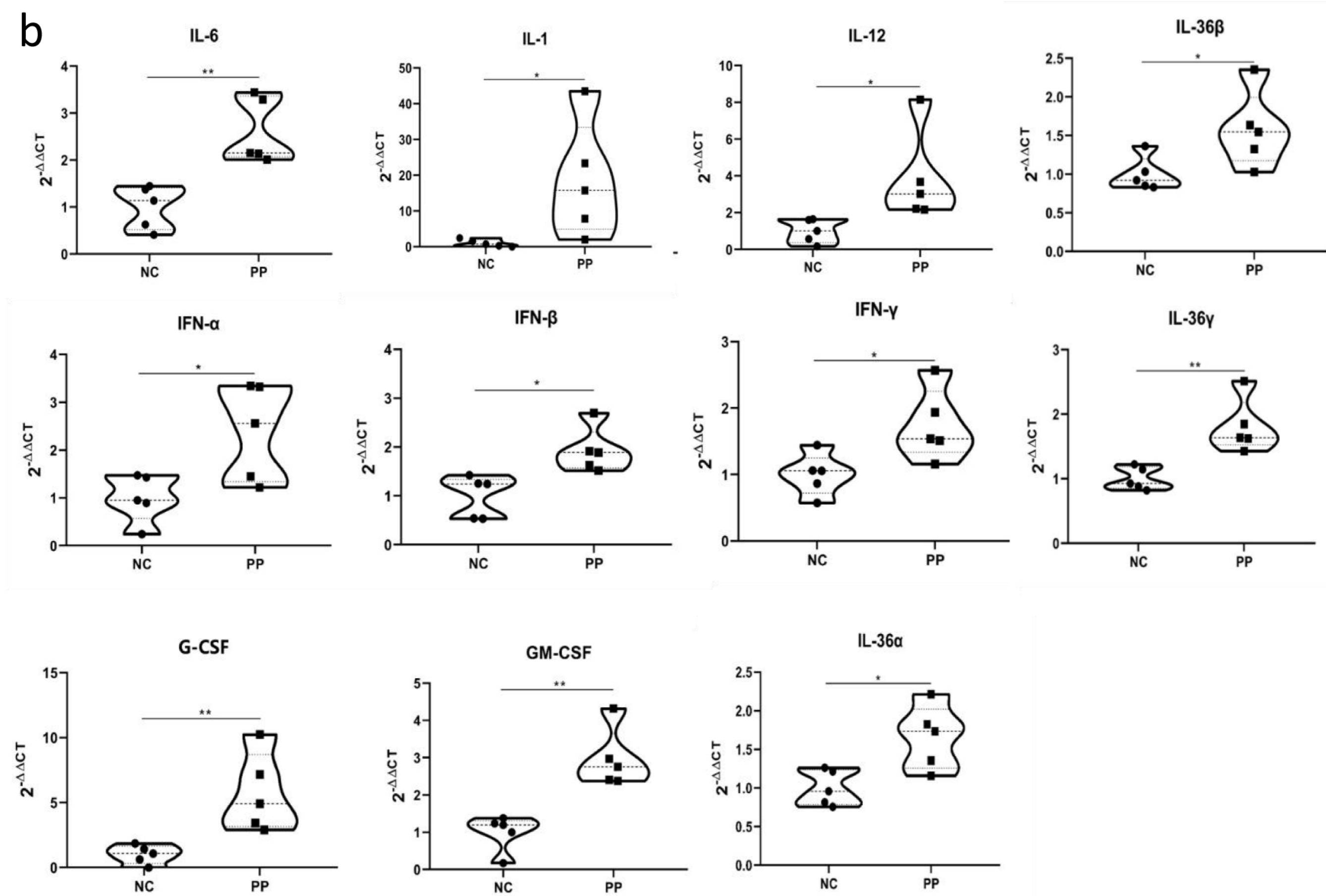

Supplement: Supplementary file 1 — Supplementary figure 1 [file 41419_2023_5815_MOESM1_ESM.pdf]

**a**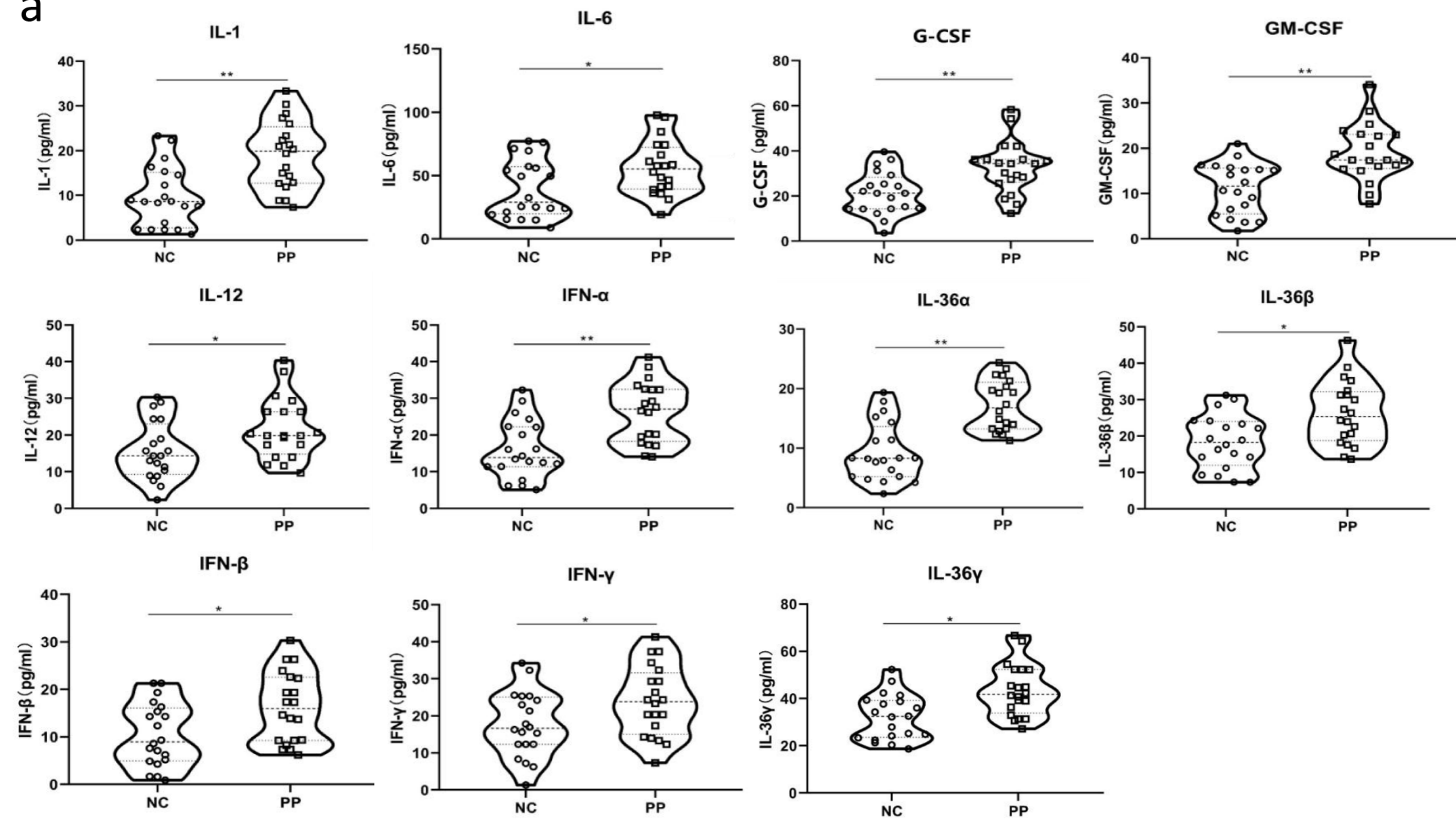**b**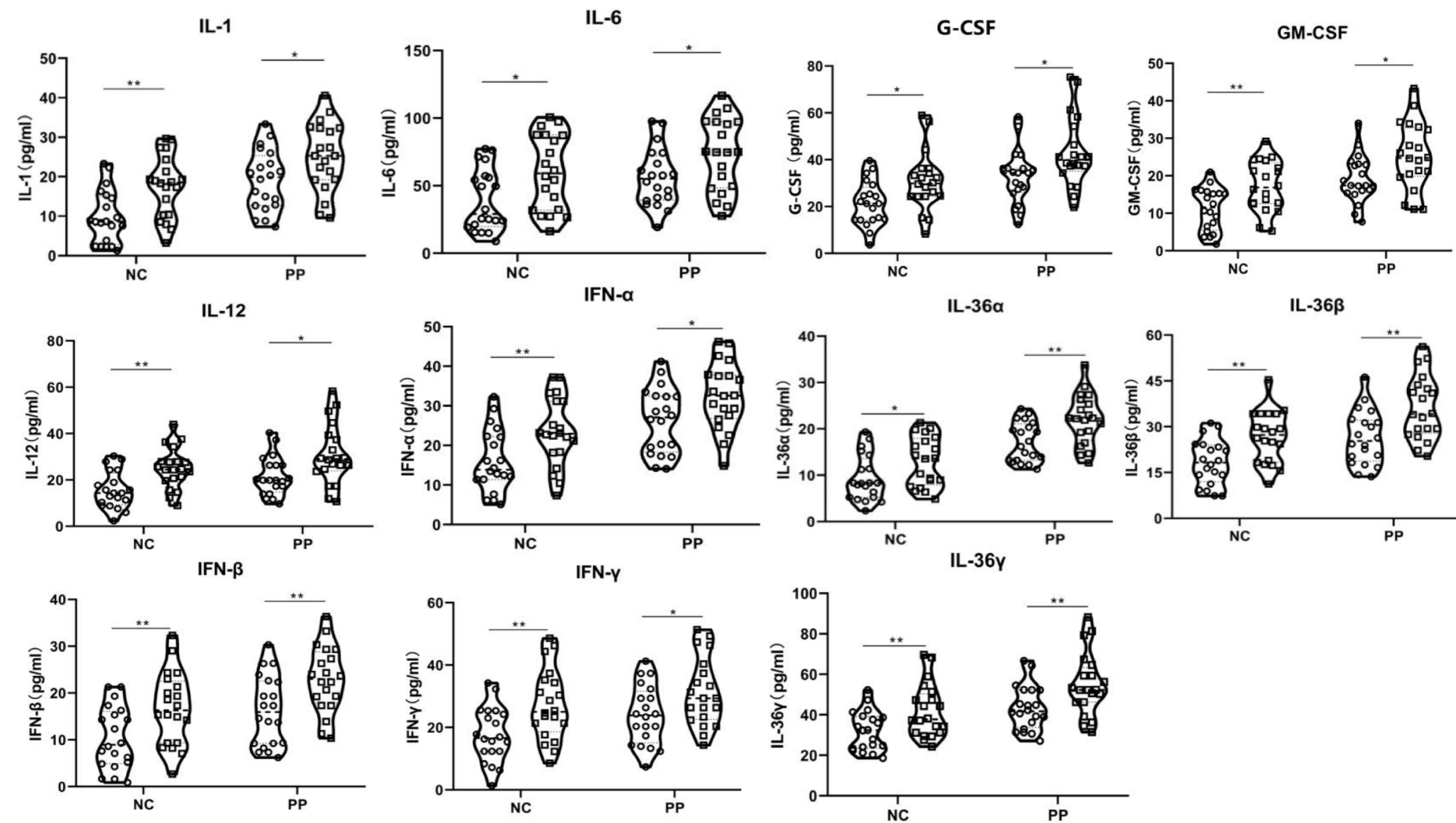

Supplement: Supplementary file 2 — Supplementary figure 2 [file 41419_2023_5815_MOESM2_ESM.pdf]

a

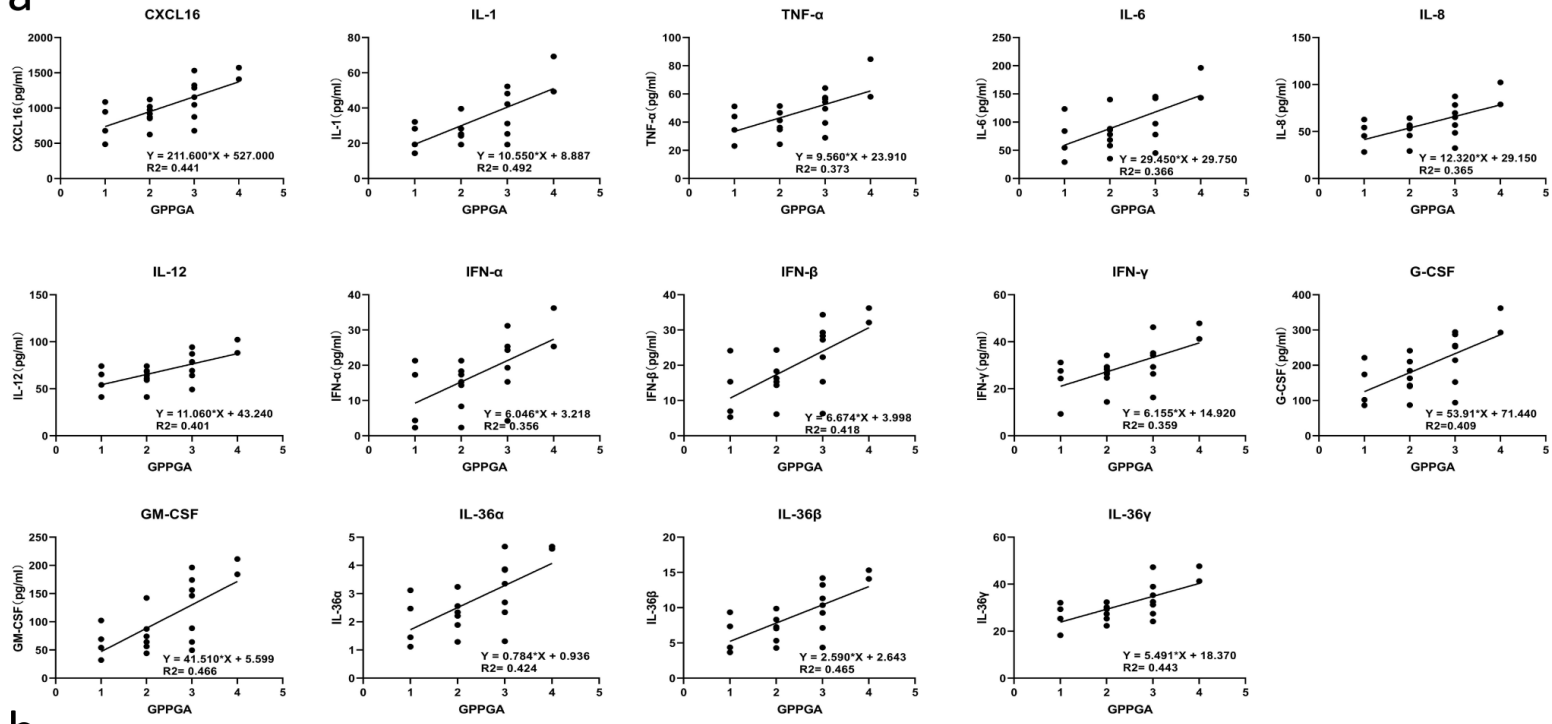

b

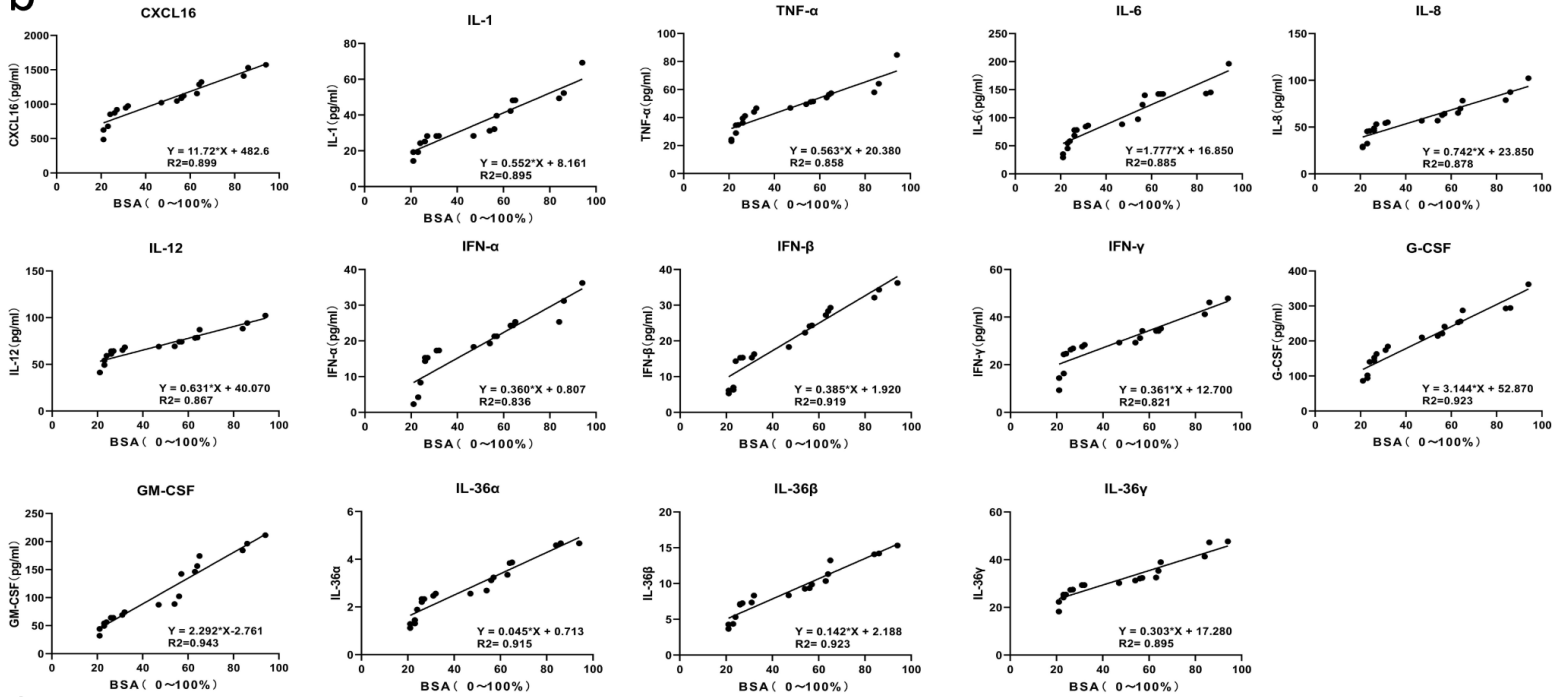

c

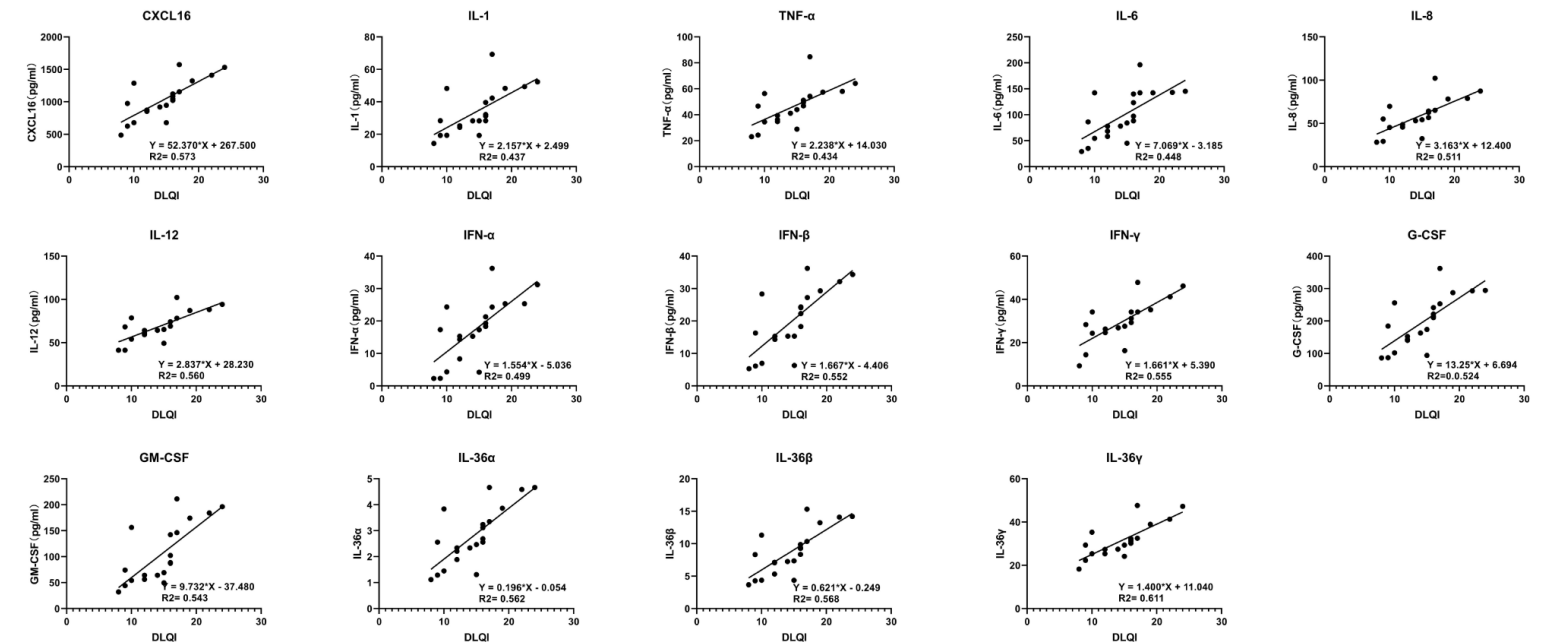

Supplement: Supplementary file 3 — Supplementary figure 3 [file 41419_2023_5815_MOESM3_ESM.pdf]

**a** *Cd11c-Cre Myd88<sup>ff</sup>*

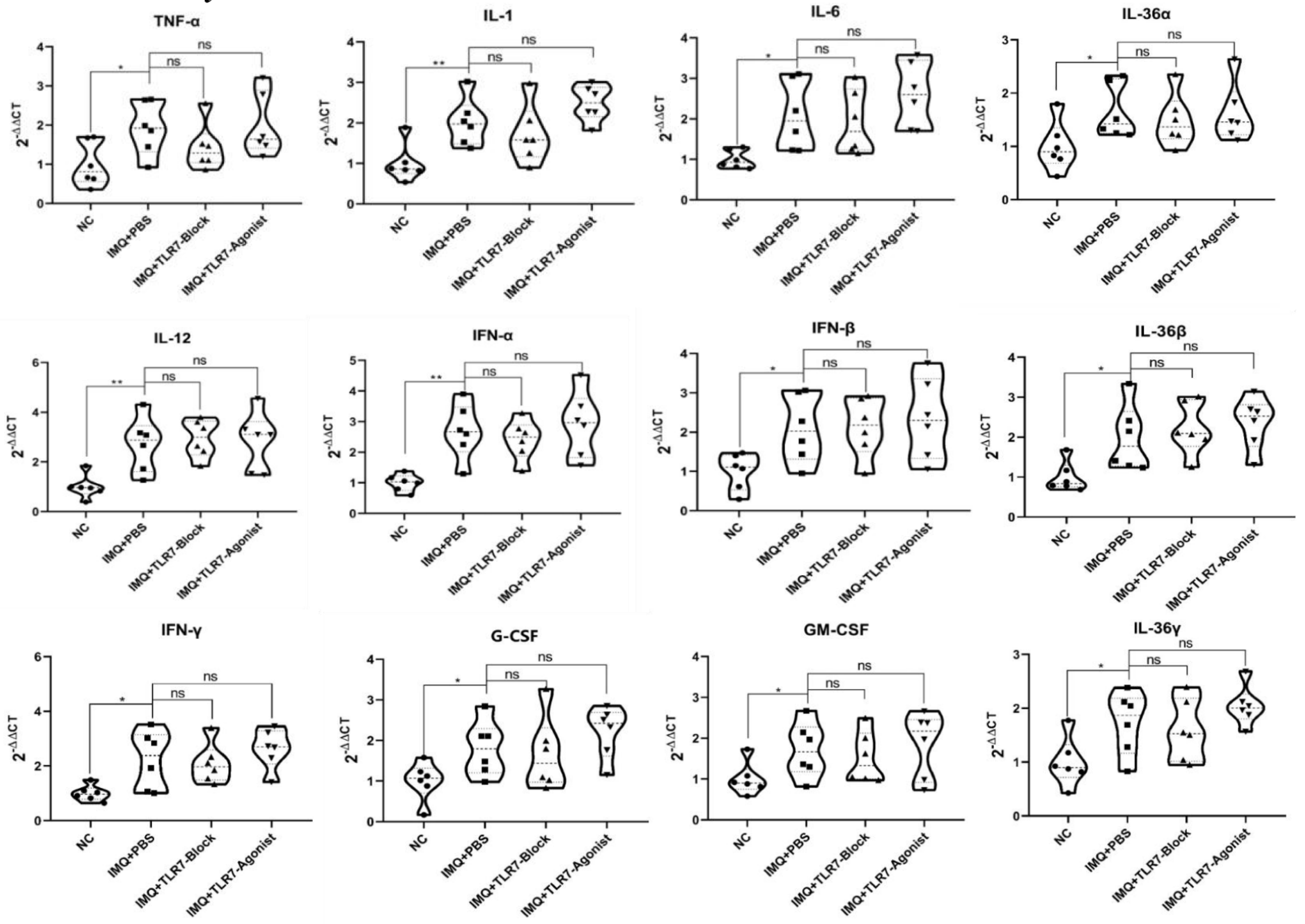

**b** *Mrp8-Cre Cxcr6<sup>ff</sup>*

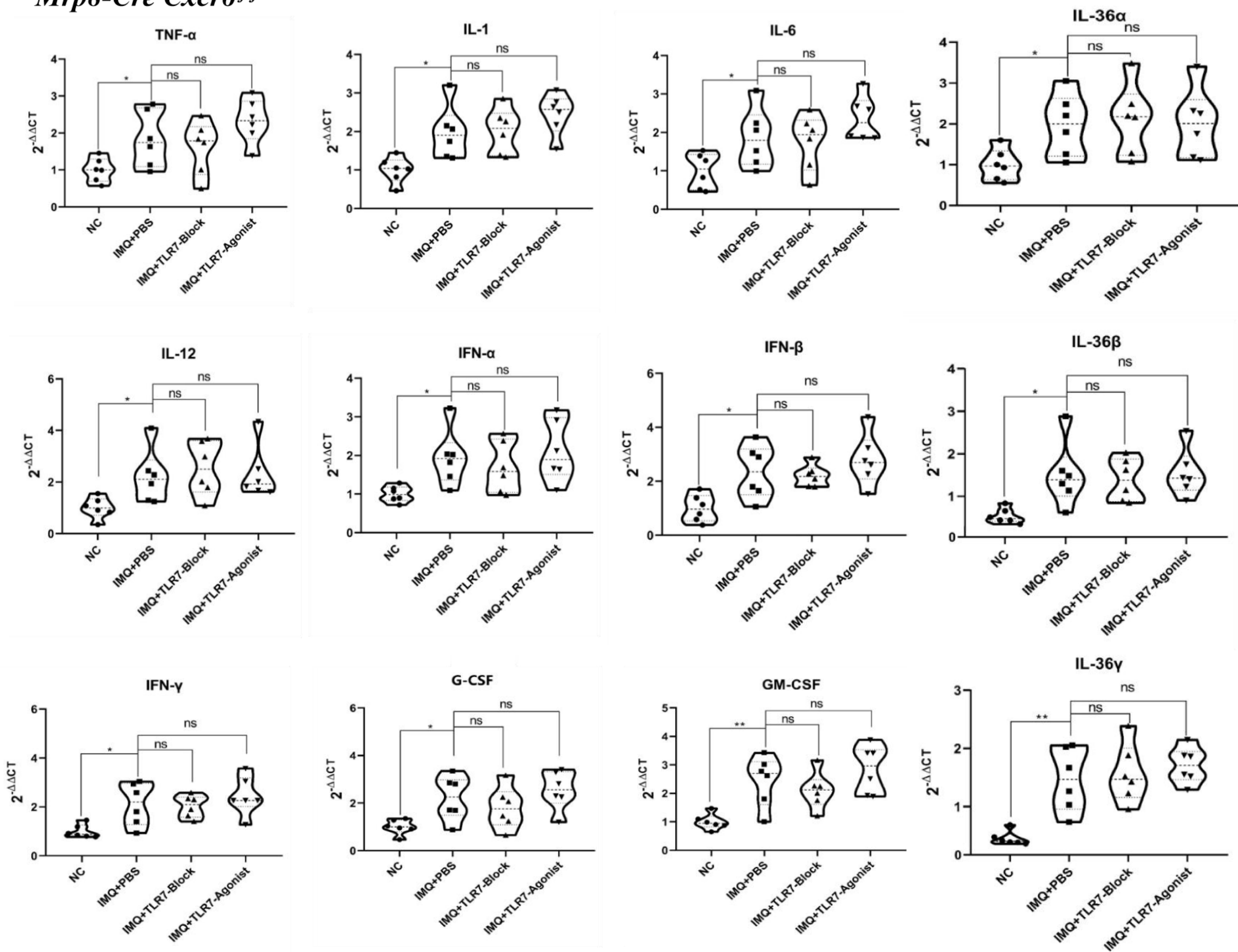

Supplement: Supplementary file 4 — Supplementary figure 4 [file 41419_2023_5815_MOESM4_ESM.pdf]

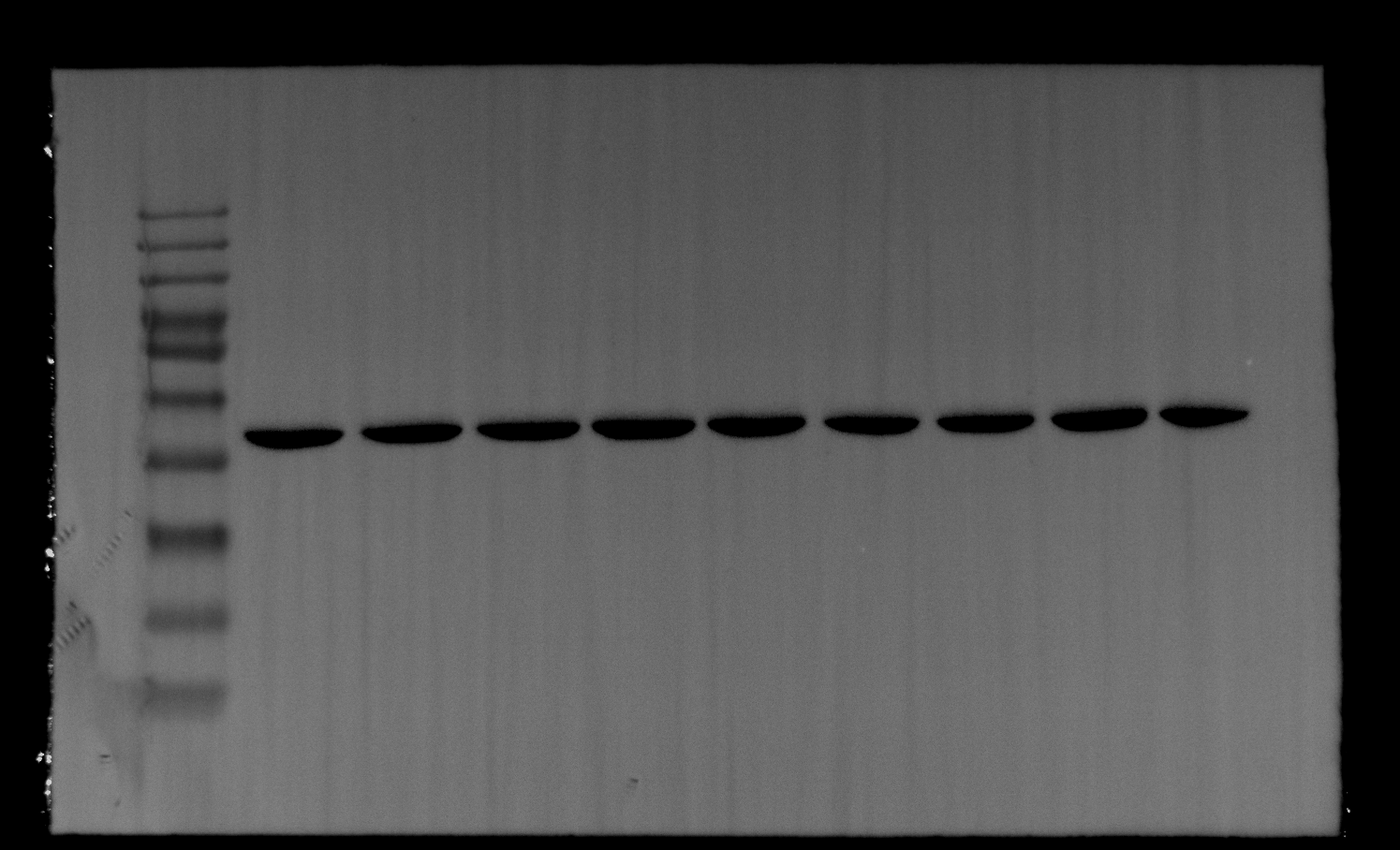

Supplement: Supplementary file 9 — orginal western blots-ACTIN [file 41419_2023_5815_MOESM9_ESM.tif]

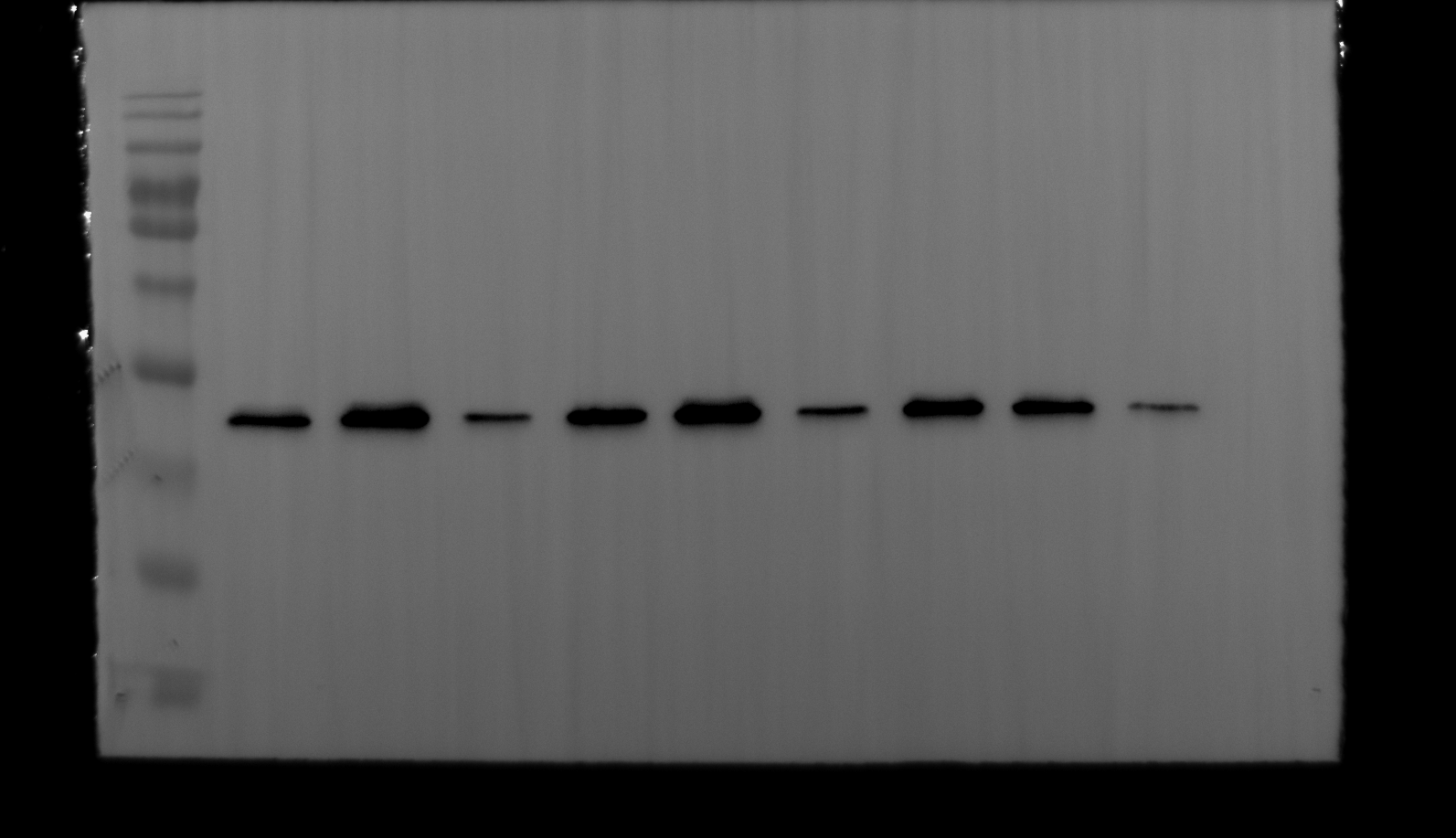

Supplement: Supplementary file 10 — orginal western blots-CXCL16 [file 41419_2023_5815_MOESM10_ESM.tif]

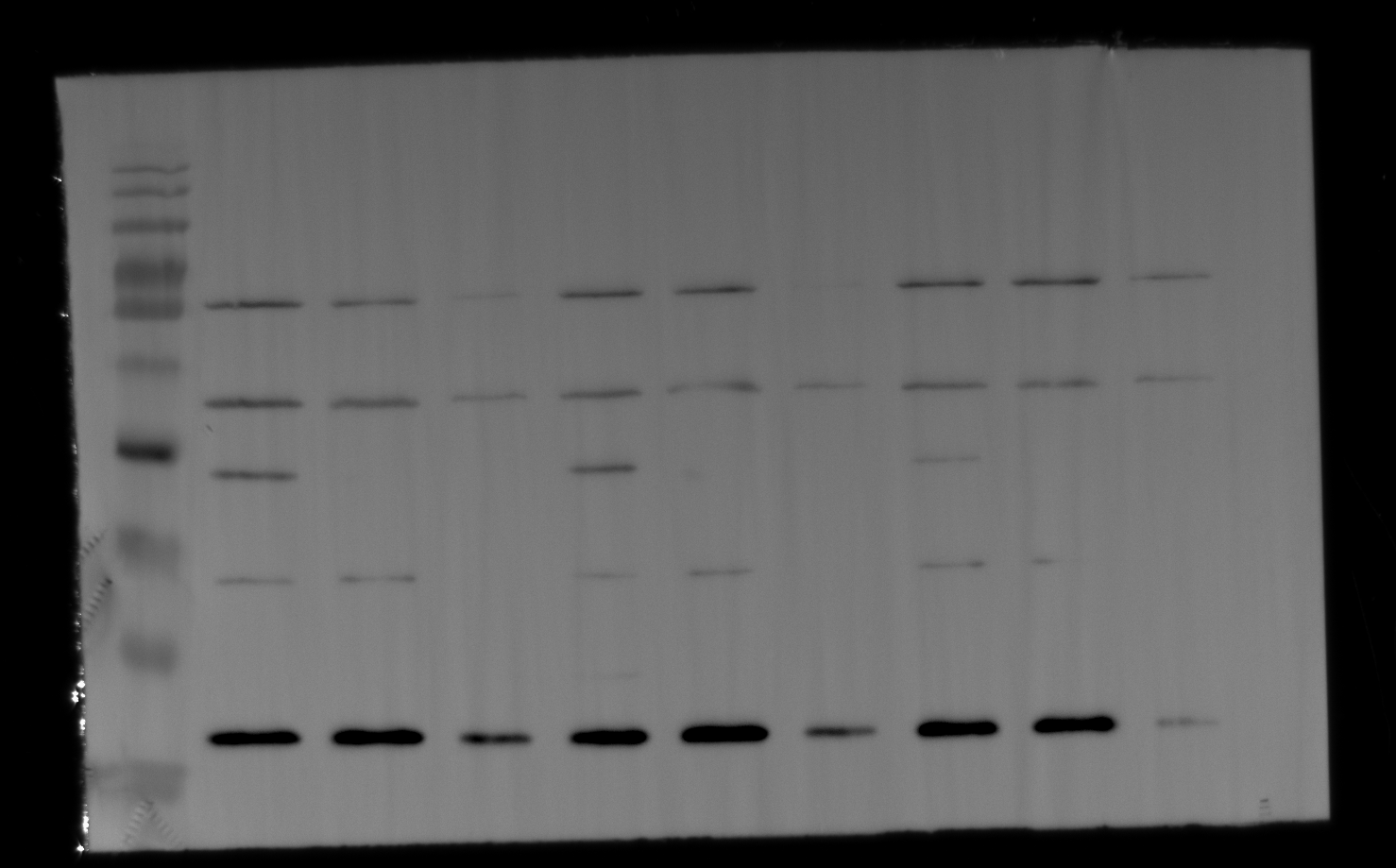

Supplement: Supplementary file 11 — orginal western blots-IL-36α [file 41419_2023_5815_MOESM11_ESM.tif]

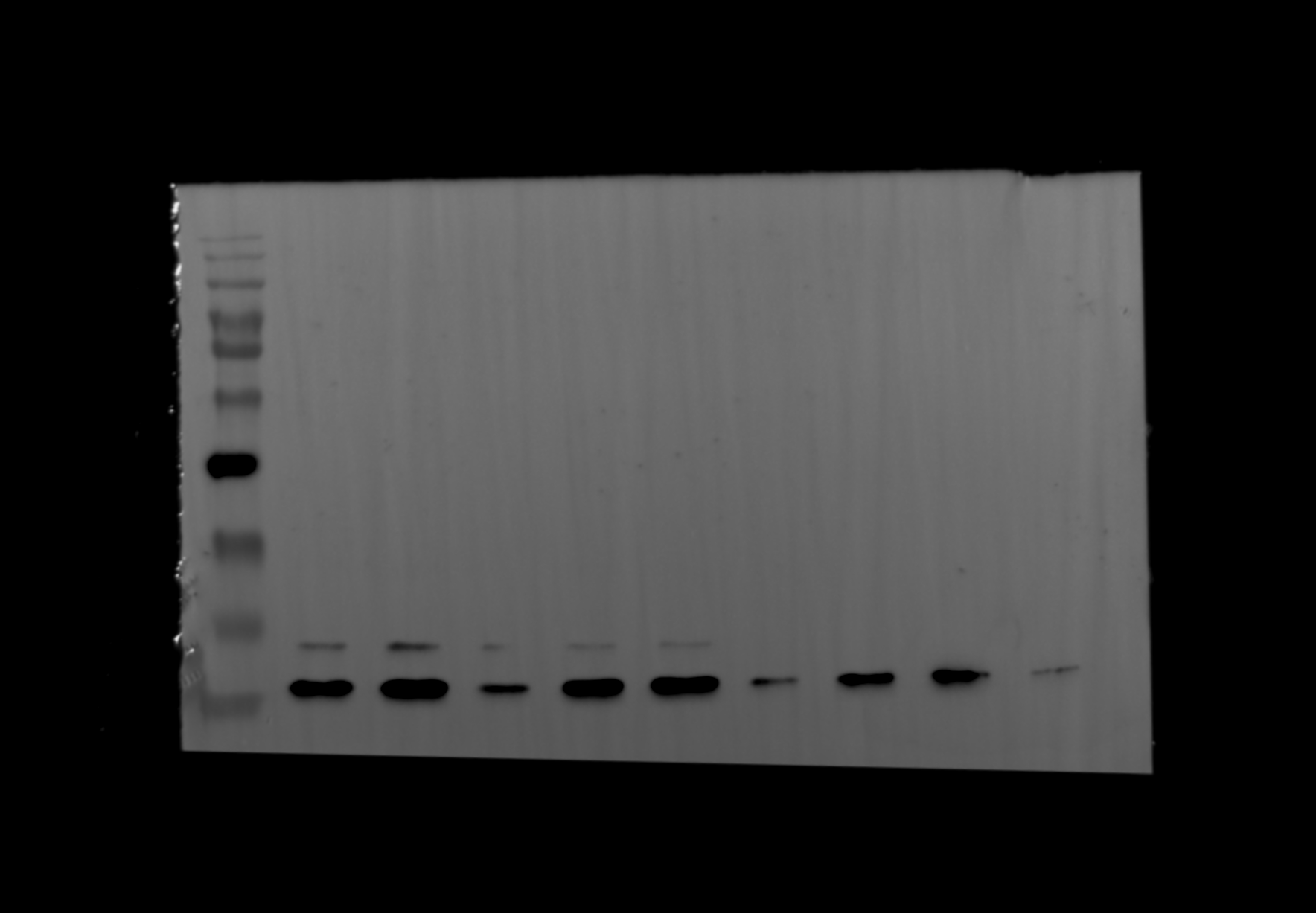

Supplement: Supplementary file 12 — orginal western blots-IL-36β [file 41419_2023_5815_MOESM12_ESM.tif]
