# Supplementary material for: TLR7-MyD88-DC-CXCL16 axis results neutrophil activation to elicit inflammatory response in pustular psoriasis
Source: Cell Death Dis. 2023 May 9;14(5):315. doi: 10.1038/s41419-023-05815-y (PMC10170143; doi:10.1038/s41419-023-05815-y)
Supplement: Supplementary file 5 — Supplementary table 1 [file 41419_2023_5815_MOESM5_ESM.docx]

Supplementary table 1. Generalized Pustular Psoriasis Area and Severity Index (GPPASI)

| Generalized Pustular Psoriasis Area and Severity Index**（GPPASI）** | | | | | | | |
| --- | --- | --- | --- | --- | --- | --- | --- |
| Score | 0 | 1 | 2 | 3 | 4 | 5 | 6 |
| Erythema（E） | None | Almost none | light | moderate | severe |  |  |
| Pustule（P） | None | Almost none | light | moderate | severe |  |  |
| Desquamation（D） | None | Almost none | light | moderate | severe |  |  |
| Proportion（%） | 0 | 1～10 | 10～30 | 30～50 | 50～70 | 70～90 | 90～100 |

GPPASI=（E+P+D）× Proportion × 0.1（head）+（E+P+D）× Proportion × 0.2（upper limbs）+（E+P+D）× Proportion × 0.3（trunk）+ (E+P+D）× Proportion × 0.4（lower limbs）
