# Supplementary material for: TLR7-MyD88-DC-CXCL16 axis results neutrophil activation to elicit inflammatory response in pustular psoriasis
Source: Cell Death Dis. 2023 May 9;14(5):315. doi: 10.1038/s41419-023-05815-y (PMC10170143; doi:10.1038/s41419-023-05815-y)
Supplement: Supplementary file 6 — Supplementary table 2 [file 41419_2023_5815_MOESM6_ESM.docx]

Supplementary table 2. Generalized Pustular Psoriasis Physician Global Assessment (GPPGA)

| Generalized Pustular Psoriasis Physician Global Assessment**（GPPGA）** | | | | | |
| --- | --- | --- | --- | --- | --- |
| Score | 0 | 1 | 2 | 3 | 4 |
|  | None | Almost none | light | moderate | severe |
| Erythema（E） | Normal or post inflammatory pigmentation | Slight erythema, light pink | Light red | Bright red | Crimson |
| Pustule（P） | No visible pustules | Scattered (no fusion) small pustules are occasionally seen | Scattered (no fusion) small pustules distributed in clusters | Densely distributed small pustules, partially fused | Densely distributed pustules fuse into a large pus |
| Desquamation（D） | None | Only local desquamation at the edge of skin lesions | Most of the lesions are slightly desquamated | Most of the lesions are moderately desquamated | Most of the lesions were severely desquamated |

GPPGA=（E+P+D）/3，Represents the average of the scores of the three components；PGA score of GPP 0=The average of all three items is 0；1 = 0 < mean value <1.5；2 = 1.5 ≤ mean value < 2.5；3 = 2.5 ≤ mean value < 3.5；4 = mean value ≥ 3.5
