# Supplementary material for: TLR7-MyD88-DC-CXCL16 axis results neutrophil activation to elicit inflammatory response in pustular psoriasis
Source: Cell Death Dis. 2023 May 9;14(5):315. doi: 10.1038/s41419-023-05815-y (PMC10170143; doi:10.1038/s41419-023-05815-y)
Supplement: Supplementary file 7 — Supplementary table 3 [file 41419_2023_5815_MOESM7_ESM.docx]

Supplementary table 3. Body Surface Area (BSA)

| Body Surface Area**（BSA）** | | | | |
| --- | --- | --- | --- | --- |
| Position | Head / neck | Upper limbs | Trunk  (including armpit and perineum) | Lower limbs  (including buttocks) |
| Proportion of total body surface area | 10 % | 20 % | 30 % | 40 % |
| Equivalent to palm area | 10 | 20 | 30 | 40 |
| The surface area of the affected body is equivalent to the number of palms |  |  |  |  |
| Total body surface area affected (BSA) | % | | | |
