# Supplementary material for: TLR7-MyD88-DC-CXCL16 axis results neutrophil activation to elicit inflammatory response in pustular psoriasis
Source: Cell Death Dis. 2023 May 9;14(5):315. doi: 10.1038/s41419-023-05815-y (PMC10170143; doi:10.1038/s41419-023-05815-y)
Supplement: Supplementary file 8 — Supplementary table 4 [file 41419_2023_5815_MOESM8_ESM.docx]

Supplementary table 4. [Dermatology Life Quality Index (DLQI)](http://www.baidu.com/link?url=NFIXtWxovOf2xj5bUmY6E4w2lT-M36L2w-E_Xhae2dEJnreyt3DCkhyaxBMNFeAztY1EVAUmSyt68Fg238qZIq)

| Dermatology Life Quality Index**（DLQI）** | | | | |
| --- | --- | --- | --- | --- |
| Score | None  （0） | slight  （1） | serious  （2） | Extremely serious（3） |
| 1. Do you have the feeling of "itching" or "pain" on your skin? |  |  |  |  |
| 2. Do you have "depression", "embarrassment", "Sadness" and other emotions due to skin problems? |  |  |  |  |
| 3. Will your skin problems affect your daily housekeeping and shopping? |  |  |  |  |
| 4. Do you choose different or special clothes and shoes due to skin problems? |  |  |  |  |
| 5. Will your skin problems affect your social, entertainment or outdoor activities? |  |  |  |  |
| 6. Do you have skin problems that affect your daily sports? |  |  |  |  |
| 7. Will your work or study be affected by skin problems? How big is the problem of your skin in your work or study? |  |  |  |  |
| 8. Will your skin problems affect your relationship with your spouse, friends and relatives? |  |  |  |  |
| 9. Do you have skin problems that affect your sex life? |  |  |  |  |
| 10. Does your skin problem cause inconvenience in daily life? |  |  |  |  |
